# Tomo-seq identifies NINJ1 as a potential target for anti-inflammatory strategy in thoracic aortic dissection

**DOI:** 10.1186/s12916-023-03077-1

**Published:** 2023-10-20

**Authors:** Yixuan Sheng, Liying Wu, Yuan Chang, Wendao Liu, Menghao Tao, Xiao Chen, Xiong Zhang, Bin Li, Ningning Zhang, Dongting Ye, Chunxi Zhang, Daliang Zhu, Haisen Zhao, Aijun Chen, Haisheng Chen, Jiangping Song

**Affiliations:** 1 Department of Cardiovascular Surgery, Guangzhou First People’s Hospital, School of Medicine, South China University of Technology, Guangzhou, China; 2 State Key Laboratory of Cardiovascular Disease, Fuwai Hospital, National Centre for Cardiovascular Diseases, Chinese Academy of Medical Sciences, Peking Union Medical College, Beijing, China; 3 First Clinical Medical College, Guangzhou University of Chinese Medicine, Guangzhou, China; 4 Present Address: Beijing Key Laboratory of Preclinical Research and Evaluation for Cardiovascular Implant Materials, Animal Experimental Centre, Fuwai Hospital, National Centre for Cardiovascular Disease, Chinese Academy of Medical Sciences and Peking Union Medical College, Beijing, 100037 China; 5 Shenzhen Key Laboratory of Cardiovascular Disease, Fuwai Hospital Chinese Academy of Medical Sciences, Shenzhen, 518057 China

**Keywords:** Thoracic aortic dissection, Aortic remodeling, Tomo-seq, Immune, NINJ1, Inflammation

## Abstract

**Background:**

Thoracic aortic dissection (TAD) is a life-threatening disease caused by an intimal tear in the aorta. The histological characteristics differ significantly between the tear area (TA) and the distant area. Previous studies have emphasized that certain specific genes tend to cluster at the TA. Obtaining a thorough understanding of the precise molecular signatures near the TA will assist in discovering therapeutic strategies for TAD.

**Methods:**

We performed a paired comparison of the pathological patterns in the TA with that in the remote area (RA). We used Tomo-seq, genome-wide transcriptional profiling with spatial resolution, to obtain gene expression signatures spanning from the TA to the RA. Samples from multiple sporadic TAD patients and animal models were used to validate our findings.

**Results:**

Pathological examination revealed that the TA of TAD exhibited more pronounced intimal hyperplasia, media degeneration, and inflammatory infiltration compared to the RA. The TA also had more apoptotic cells and CD31^+^α-SMA^+^ cells. Tomo-seq revealed four distinct gene expression patterns from the TA to the RA, which were inflammation, collagen catabolism, extracellular matrix remodeling, and cell stress, respectively. The spatial distribution of genes allowed us to identify genes that were potentially relevant with TAD. NINJ1 encoded the protein-mediated cytoplasmic membrane rupture, regulated tissue remodeling, showed high expression levels in the tear area, and co-expressed within the inflammatory pattern. The use of short hairpin RNA to reduce NINJ1 expression in the beta-aminopropionitrile-induced TAD model led to a significant decrease in TAD formation. Additionally, it resulted in reduced infiltration of inflammatory cells and a decrease in the number of CD31^+^α-SMA^+^ cells. The NINJ1-neutralizing antibody also demonstrated comparable therapeutic effects and can effectively impede the formation of TAD.

**Conclusions:**

Our study showed that Tomo-seq had the advantage of obtaining spatial expression information of TAD across the TA and the RA. We pointed out that NINJ1 may be involved in inflammation and tissue remodeling, which played an important role in the formation of TAD. NINJ1 may serve as a potential therapeutic target for TAD.

**Supplementary Information:**

The online version contains supplementary material available at 10.1186/s12916-023-03077-1.

## Background

Thoracic aortic dissection (TAD) is a fatal cardiovascular disease with rapid progression, high mortality, poor prognosis, and increasing incidence rate [1]. TAD is characterized by the rapid development of the intimal flap, which can extend both forward and backward from the site where the intima tears up [1]. Aortic remodeling, which involves alterations in the structure and function of blood vessels, is a histological characteristic of TAD that exhibits regional and heterogeneous variations [1–4]. The spatial expression distribution and function of genes associated with aortic remodeling are crucial for understanding the pathophysiology of TAD [5]. Studies have suggested that the spatial distribution of inflammatory-related markers tends to gradually increase from the intact area to the border area and the tear area (TA) [6]. Intimal tear has been suggested as the initiating event in TAD [1], but the underlying molecular changes of the site of the tearing up remain poorly characterized. It is crucial to elucidate the initiating mechanisms that trigger the tearing or rupture of the aorta.

Previous studies have consistently relied on tissues extracted from various individuals, resulting in discernible individual variations. Meanwhile, traditional transcriptomes from whole-tissue homogenates mix varying levels of lesion information and lack the spatial resolution to accurately reflect the true situation [7, 8]. Tomography RNA sequencing (Tomo-seq), a spatial resolution transcription method proposed in 2014, provides continuous changes in gene expression from diseased areas to relatively normal areas based on continuous cryosections of specific regions [9–12]. Our histology examination confirmed the pathological differences along the tear and remote area in TAD. We performed Tomo-seq to identify the spatial gene expression signatures along the tear and remote area in TAD. For the first time, spatial distribution patterns in gene expression along the tear and remote area have been observed. *NINJ1*, encoding Ninjurin 1, had expression peaks in the TA. NINJ1 is a homophilic transmembrane adhesion molecule involved in various processes of tissue remodeling such as inflammation and cell death [13–15]. In our study, NINJ1 exhibited a similar trend to the genes associated with inflammatory responses. Moreover, we also noticed that NINJ1 co-localized with various cell death markers, including GSDMD, HMGB1, and TUNEL. Inhibition of NINJ1 by short hairpin RNA (shRNA) and neutralizing antibody delayed the development of beta-aminopropionitrile (BAPN)-induced TAD and reduced overall mortality. Our results indicated that NINJ1 may be associated with the formation of TAD. Tomo-seq could be used to increase our understanding of the mechanisms of vascular remodeling, which could help facilitate the development of effective therapeutics for TAD.

## Methods

### Patient specimens collection

The aorta tissues of TAD were obtained from eight patients diagnosed with sporadic TAD who underwent the aortic repair surgery operated within 24 h of onset. The study was approved by the Human Ethics Committee of Guangzhou First People’s Hospital (K-2019–156-02). Written informed consent was obtained from each patient. Patients with aortic disease triggered by gene mutation, such as Marfan’s syndrome, or associated with bicuspid aortic valves were excluded based on clinical diagnosis. All samples were collected within 30 min after aorta excision. Samples were repeatedly flushed with saline at 4 °C to remove blood and mural thrombus adhering to the aortic wall, and photographs recorded the tear boundary. Afterward, these samples were cut according to the needs of the study (Additional file 1: Fig. S1).

### Tomo-seq

Tomo-seq is a spatial resolution transcription method based on continuous sections of the target area of frozen tissues, followed by RNA extraction and sequencing from individual sections [9, 10, 16]. In short, 4-mm wide portions of human TAD tissue spanning from the tear to the remote area were embedded in a tissue-freezing medium, frozen on dry ice, and cryosectioned into 20 continuous slices of 5 μm thickness into an Eppendorf tube, 30 tubes in total. After the total RNA extraction from continuous cryosections in an individual Eppendorf tube and DNase treatment, magnetic beads with Oligo (dT) were used to isolate mRNA. The mRNA was fragmented into short fragments. Then cDNA is synthesized using the mRNA fragments as templates. Short fragments were purified and resolved with EB buffer for end reparation and single nucleotide A (adenine) addition. After that, the short fragments were connected with adapters. During the QC steps, Agilent 2100 Bioanalyzer and ABI StepOnePlus Real-Time PCR System were used to quantify and qualify the sample library. At last, the library could be sequenced using BGISEQ-500. Tomo-seq data analysis was referred to as reported by Wu et al. [11]. Fastq data were aligned to human genome hg38 using STAR (v2.5.3a), and then gene expression TPM (transcript per million) was calculated using RSEM (v1.2.31). We used GENCODE v31 as gene annotation. Genes expressed in at least three sections were selected for downstream analysis. We performed Tomo-seq data analysis using R and Bioconductor package tomoda (https://doi.org/doi:10.18129/B9.bioc.tomoda). The procedures of analysis were adapted from Wu et al. [16]. In brief, we scale the TPM of all genes across sections to obtain *Z* scores (i.e., the TPM of a gene in all sections minus the mean value divided by the standard deviation). For correlation analysis, Pearson correlation coefficients between any two sections were calculated based on the *Z* score of all genes. Next, we performed hierarchical clustering of sections using the TPM of all genes to find the borders of different zones. We found highly expressed (*Z* score > 1) genes in at least four consecutive sections and calculated the statistical significance using permutation tests to identify locally expressed genes. Almost all (337 out of 339) identified locally expressed genes are significant at *p*-value < 0.05. Then we analyzed the expression pattern in sections of identified locally expressed genes. The similarity of expression patterns among genes was measured using both dimensional reductions with t-SNE and correlation analysis. Both methods showed four groups of genes that were locally expressed in different sections. The plots of gene TPM changes across sections were locally smoothed with LOESS.

### Statistical analysis

Values are presented as mean ± standard error of the mean (SEM). Statistical analyses between two groups were conducted using the two-tailed unpaired or paired Student’s *t-*test. Comparison among > two groups was performed using one-way ANOVA analysis. For classified data, the chi-square test was used. Pearson’s correlation coefficients were used to calculate gene pair correlation based on gene expression in human samples. Gene ontology (GO)-term analysis on ranked lists was performed using the online database Metascape. *P* value < 0.05 was interpreted to denote statistical significance. All the statistical analysis methods were indicated in the corresponding figure legends. GraphPad Prism 8.0.1 software (San Diego, CA, USA) was used for statistical analyses.

### Histological analysis

The aortas were fixed in 10% formalin, embedded in paraffin, and cut into 5-μm-thick sections. The hematoxylin and eosin (H&E), Verhoeff’s Van Gieson (EVG) staining (G1597, Solarbio), and Masson’s trichrome staining (G1340, Solarbio) were performed on the paraffin sections of the aorta according to the kit instructions and finally observed with a pathologic scanner (Zeiss, Oberkochen, Germany). The elastin degradation was graded on a scale of 1–4, where 1 for < 25% degradation, 2 for 25–50% degradation, 3 for 50–75% degradation, and 4 for > 75% degradation, dissection, or rupture [17]. Quantitative analysis of related positive area and number of cells in tissues (the ratio of a total number of positive points /the area of the entire section) using Image-Fiji.

### Immunohistochemistry (IHC)

Antigen retrieval was performed on the paraffin sections using ethylenediaminetetraacetic acid (EDTA) solution (pH 9.0, ZLI-9068, ZSBG-BIO, China). The endogenous peroxidase activity was blocked by 3% hydrogen peroxide for 15 min after being washed in PBS, and nonspecific binding was blocked using goat serum (ZLI-9056, ZSBG-BIO, China) for 45 min at room temperature. Sections were stained with the primary antibodies overnight at 4 °C. After washing 3 times with PBS, the slides were incubated with appropriate HRP-labeled secondary antibodies for 20 min at room temperature and then performed chromogenic DAB staining. The positive area (%) of NINJ1 was determined utilizing the Image J IHC Toolbox plugin. In short, the overall area of NINJ1-positive pixels is computed and divided by the overall area of the tissue contained within the section to acquire the NINJ1 area ratio.

### Multiple immunostaining

The Opal 7 multiplexed assay (PerkinElmer, MA, USA) was used to generate multiple immunostainings. The best concentration of antibodies was determined before multiple immunostainings, according to the instructions. The primary antibodies used were as follows: anti-CD45 (1:200, ab10558, Abcam), anti-α-SMA (1:2000, ab12964, Abcam), anti-VIM (1:150, ab8978, Abcam), anti-CD31 (1:50,ab9498,Abcam), anti-NINJ1 (1:200, GTX31596, GeneTex), anti-TPPP3 (1:200, GTX33554, GeneTex), anti-CD3 (1:200, 17,617–1-P,Thermo), anti-MMP9 (1:1000, ab74003,Abcam), anti-CD11b (1:3000, ab133357, Abcam), anti-CD31 (1:50, ab28364,Abcam), anti-GSDMD (1:800, 36,425, Cell signaling), anti-HMGB1(1:500, ab79823, Abcam). The stained slides were analyzed by Vectra Polaris Quantitative Pathology Imaging Systems (PerkinElmer).

### TUNEL apoptosis assay

Paraffin-embedded aortic sections were stained with the One Step TUNEL Apoptosis Assay Kit A (C1090, Beyotime) and counterstained with hematoxylin according to the manufacturer’s recommendation.

### Mouse TAA/TAD model

Wildtype male C57BL/6J were used for this study. Animals were purchased from the Charles River Laboratories (Beijing, China) and approved by the Animal Ethics Committee at Fuwai Hospital (FW-2022–0015). Animal experiments were designed according to The ARRIVE guidelines 2.0 (Additional file 2) [18]. All mice were maintained in IVCs at the density of 3–5 mice per cage in an SPF animal room with temperature-controlled (23 ± 2 °C), humidity of 50 ± 5%, and a dark/light cycle of 12 h. Age- and weight-matched mice were randomized into different groups by random number table method. Three-week-old male C57BL/6 mice were fed a normal diet and administered freshly prepared BAPN (Sigma-Aldrich, USA) solution dissolved in the drinking water (1g/kg/day) for consecutive 4 weeks to establish the TAA (Thoracic aortic aneurysm)/TAD model as previously described [19–21]. The same-aged mice fed with a regular diet and drinking water were served as controls. All mice that died before the expected end time of the experiment were autopsied immediately to confirm whether they died of aortic rupture, we excluded mice that did not die from TAD. The aorta of mice was examined by ultrasonic examination at 3 weeks after BAPN induction. Mice that survived for 4 weeks were sacrificed using an overdose of sodium pentobarbital, and their thoracic aortic tissue was collected for further analysis. TAA is defined as arterial dilation to more than 50% of the normal diameter. TAD is characterized by the formation of a false lumen with the blood in the medial layer, or massive blood clots in the thoracic cavity. Mouse feeding, conduction of the TAA/TAD model, and phenotype identification are handled by different individuals, and the person responsible for phenotype identification is not aware of the group information in advance. The minimum number of mice in each group was not less than six.

### Adeno-associated virus (AAV) and NINJ1 knockdown

Ninj1 shRNA fragment was cloned into adeno-associated virus 9 (AAV9) vector (pAV-U6-shRNA-CMV-GFP) (WZ Biosciences Inc.) to construct AAV9-shNinj1. Three-week-old male C57BL/6 mice were injected with 20 μl of either 5.0 × 10^11^ v.g AAV-shNinj1(Ninj1-shRNA group, *n* = 10) and with AAV-NC (Vehicle group, *n* = 10) via retrobulbar vein. Seven days after the pretreatment, we constructed the BAPN-induced TAA/TAD mice model. Mice given the same dose of BPAN alone at the same time were used as the model group (*n* = 12) and the same aged mice fed with regular diet and drinking water for 4 weeks were served as controls (*n* = 6). The above experiments were all conducted on mice of the same age and subjected to different treatments during the same period.

### NINJ1-neutralization antibody treatment

Three-week-old male C57BL/6 mice were administered NINJ1-neutralizing antibody (2.5 μg/20 μL, BD Biosciences) (anti-NINJ1 group, *n* = 12) or with mouse IgG antibody (IgG group, *n* = 10) as control by retrobulbar vein injection at 4 days before the onset of TAA/TAD induction. Mice given the same dose of BPAN alone at the same time were used as the model group (*n* = 12) and the same aged mice fed with regular diet and drinking water for 4 weeks were served as controls (*n* = 6). The above experiments were all conducted on mice of the same age and subjected to different treatments during the same period.

### Phenyl-β-d-glucopyranoside (PDG) treatment

PDG (292,710, Sigma; CAS number: 24857607) was purchased from Merck. Forty-three-week-old male C57BL/6 mice were randomized into three groups: the normal group (*n* = 6), the model group (*n* = 24) received freshly prepared BAPN (Sigma-Aldrich, USA) solution dissolved in the drinking water (1 g/kg/day) for 4 weeks, and the PDG group (*n* = 10) that received BAPN with the same way as the model mice and daily injected intraperitoneally with PDG (100 mg/kg/day) for 3 weeks. The above experiments were all conducted on mice of the same age and subjected to different treatments during the same period.

### Quantitative RT-PCR

Total RNA was extracted from tissues using TRIzol reagent (Thermo Fisher Scientific, Carlsbad, CA), followed by isopropyl alcohol precipitation. Quantitative RT-PCR was performed using the Q5 Real-Time PCR System (Applied Biosystems, Foster City, CA) with SYBR Green Master Mix (Takara). The expression levels of the target genes were normalized against GAPDH by 2^(-△△Ct) method.

### Western blot (WB)

Aorta tissue was ground into fine powder under liquid nitrogen freezing, and proteins were extracted by RPIA containing PMSF. Primary antibodies against NINJ1 (1:200, sc-136295, Santa Cruz) and GAPDH (1:10,000, KC5G5, Aksomics) were utilized and left to incubate overnight at 4°C. Bands were observed using Pierce™ ECL peroxidase substrate (Thermo Scientific, 32,209), and then images were taken using the Tanon chemiluminescent imaging system.

## Results

### Gradient pathological changes in TAD

**Fig. 1:**
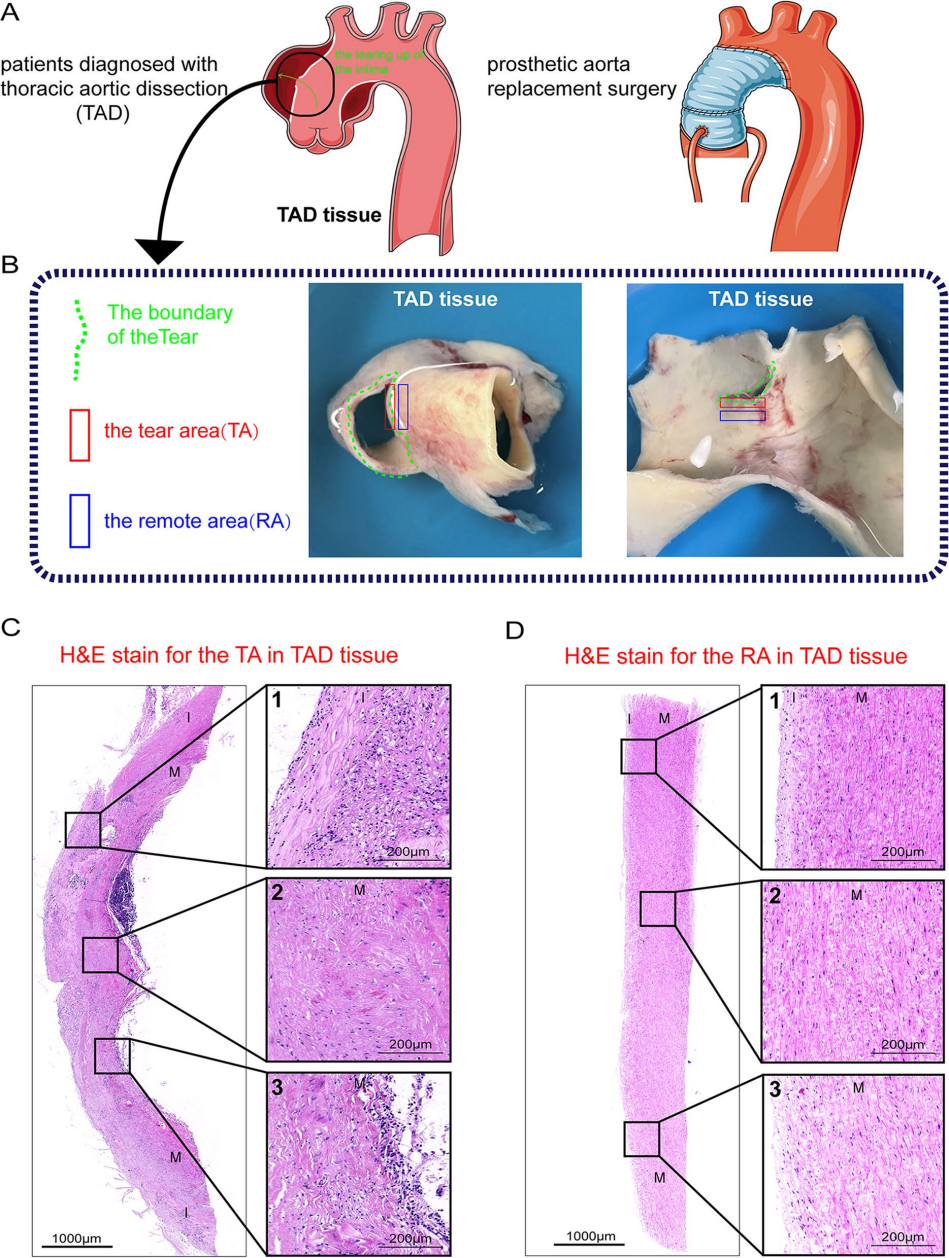
Histological examination of the TA and the RA from human TAD tissues. **A** Schematic diagram of obtaining human TAD tissue. **B** Schematic diagram of obtaining the TA and the RA from human TAD tissues. **C** H&E staining for the TA. **D** H&E staining for the RA. TA, tear area; RA, remote area. I, intimal layer; M, media layer; (1) the histological characteristics of the neointima; (2) the histological characteristics of the middle media; (3) the histological characteristics of the outer media layers

The clinical statistics of patients with TAD were listed in Additional file 3: Table S1. We obtained the TAD tissues from the patients undergoing aorta replacement surgery, as is shown in Fig. 1A, B. We performed a paired comparison of the pathological patterns in the TA and the remote area (RA) (Fig. 1C, D, Additional file 1: Fig. S2). H&E stain showed parts of the aortic wall had been exfoliated in the TA (Fig. 1C). Detailed observations clarified that significant tissue remodeling occurred in both the tunica intima and tunica media (Fig. 1C). Severe neointimal hyperplasia was present in the TA (Fig. 1C1). Disorganization of vascular smooth muscle cells and fracture of elastic fibers were observed in the tunica media (Fig. 1C2–3). In contrast, the RA had mild remodeling, characterized by a thin intima layer (Fig. 1D1) and relatively intact media with parallel fibers (Fig. 1D2–3).

**Fig. 2:**
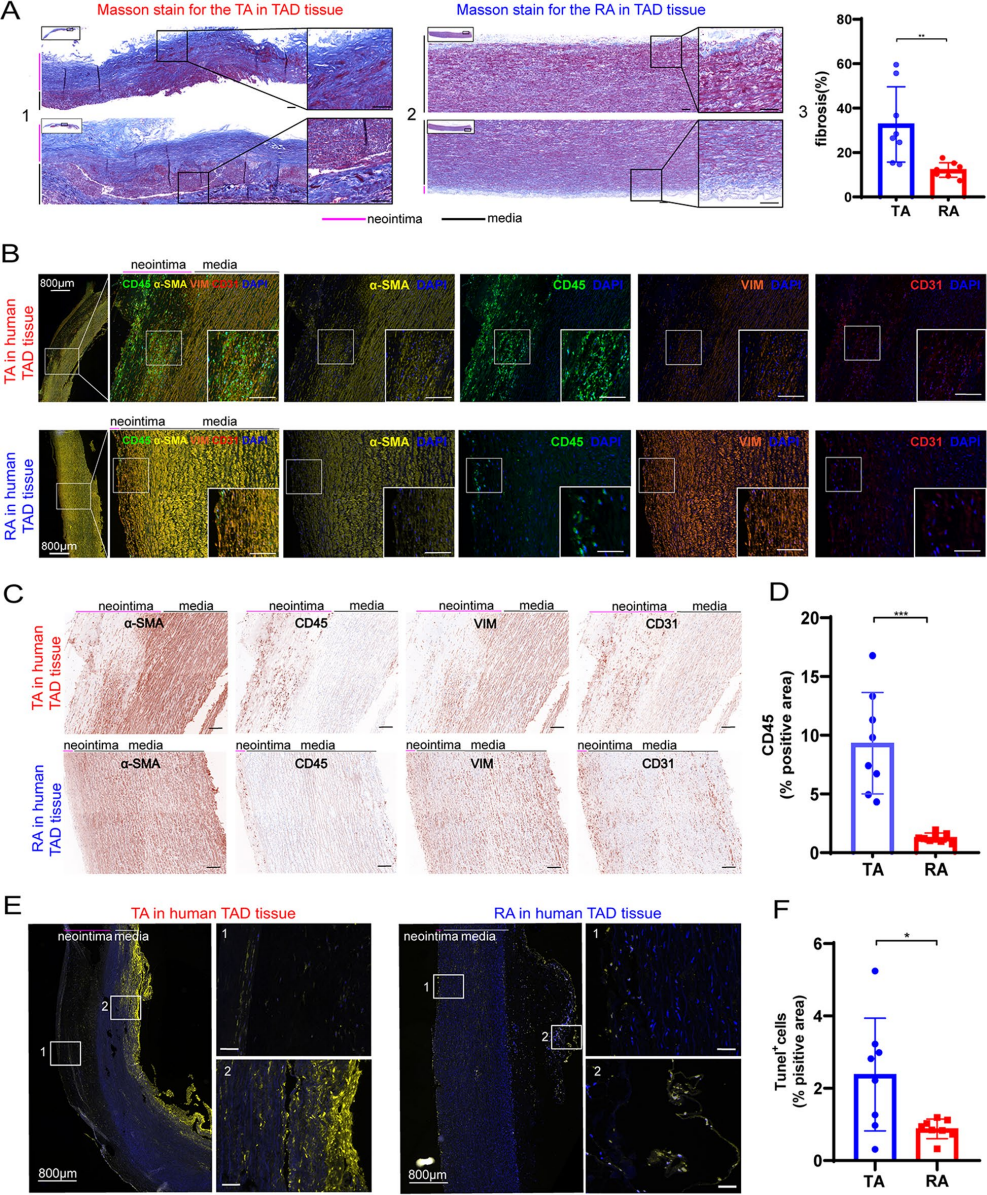
Validation of pathological features of tissue remodeling in TAD. **A** Representative photographs of Masson staining for the TA (1) and the RA (2), and quantitative analysis of aortic fibrosis area (3). **B** Representative images of immunostaining for α-SMA co-localized with CD45, VIM, and CD31 in the TA and the RA. **C** Representative images of immunostaining for α-SMA, CD45, VIM, and CD31 in the TA and the RA. **D** The quantitative analysis of CD45 infiltration in the TA and the RA. **E** Tunel staining of TA and RA of TAD. 1, the tunel stain in the neointima; 2, the tunel staining in the outer media. **F** Comparison of tunnel positive area ratio between TA and RA. TA, tear area; RA, remote area; inset in **A**, **B**, **C**, and **E** shows an enlarged local image, scale bar indicates 100 μm; *N* = 8 for each group in **A**, **D**, and **F**; *P < 0.05, **P < 0.01, ***P < 0.001; quantitative analysis was calculated as the ratio of positive area /total area of tissue section; quantitative data were shown as mean ± SEM; statistical analysis was performed with Student’s *t* test (**A**, **D**, **F**)

We performed Masson staining to clarify the gradient difference of TAD pathologies. Figure 2A1–2 showed that fibrosis was typically observed in the neointima and the outer media. Quantitative analyses revealed that the ratio of tissue fibrosis was significantly greater in the TA when compared to the RA (TA vs RA, 32.70 ± 14.96% vs 12.16 ± 2.90%, *p* = 0.0047, Fig. 2A3). To assess the differences in vascular smooth muscle cells, immune cells, fibroblasts, and endothelial cells between TA and RA. Multilabel immunofluorescence was performed to detect α-smooth muscle actin (α-SMA), CD45, Vimentin (VIM), and CD31, which correspond to the classical markers of these four cell types (Fig. 2B). We observed that α-SMA, CD45, VIM, and CD31 were positive in the neointima of both the TA and RA, but the severity was greater in TA. We also found the co-staining of CD31 and α-SMA in TAD, which is indicative of the endothelial-to-mesenchymal transition (EndMT) phenomenon (Fig. 2B). EndMT has been identified as a critical driver of vascular inflammation and fibrosis [22, 23]. The above suggested that EndMT may also be associated with TAD. IHC results also showed α-SMA, CD45, VIM, and CD31 were accumulated in the neointima region of the TA. The area ratio of CD45^+^ immune cells was enriched in the TA (TA vs RA, 9.33 ± 3.81% vs 1.29 ± 0.34%, *p* = 0.0001, Fig. 2C, D). These indicated that the pathologic changes in TA included focal smooth muscle cell hyperplasia, inflammation, and abnormal proliferation of endothelial cells, respectively. Moreover, Tunel staining showed that the TA had more apoptotic cells compared to RA (*p* = 0.0178, Fig. 2E, F). These results confirmed a longitudinal gradient of aortic pathologies from TA to RA.

### Tomo-seq on the aorta with dissection lesions

**Fig. 3:**
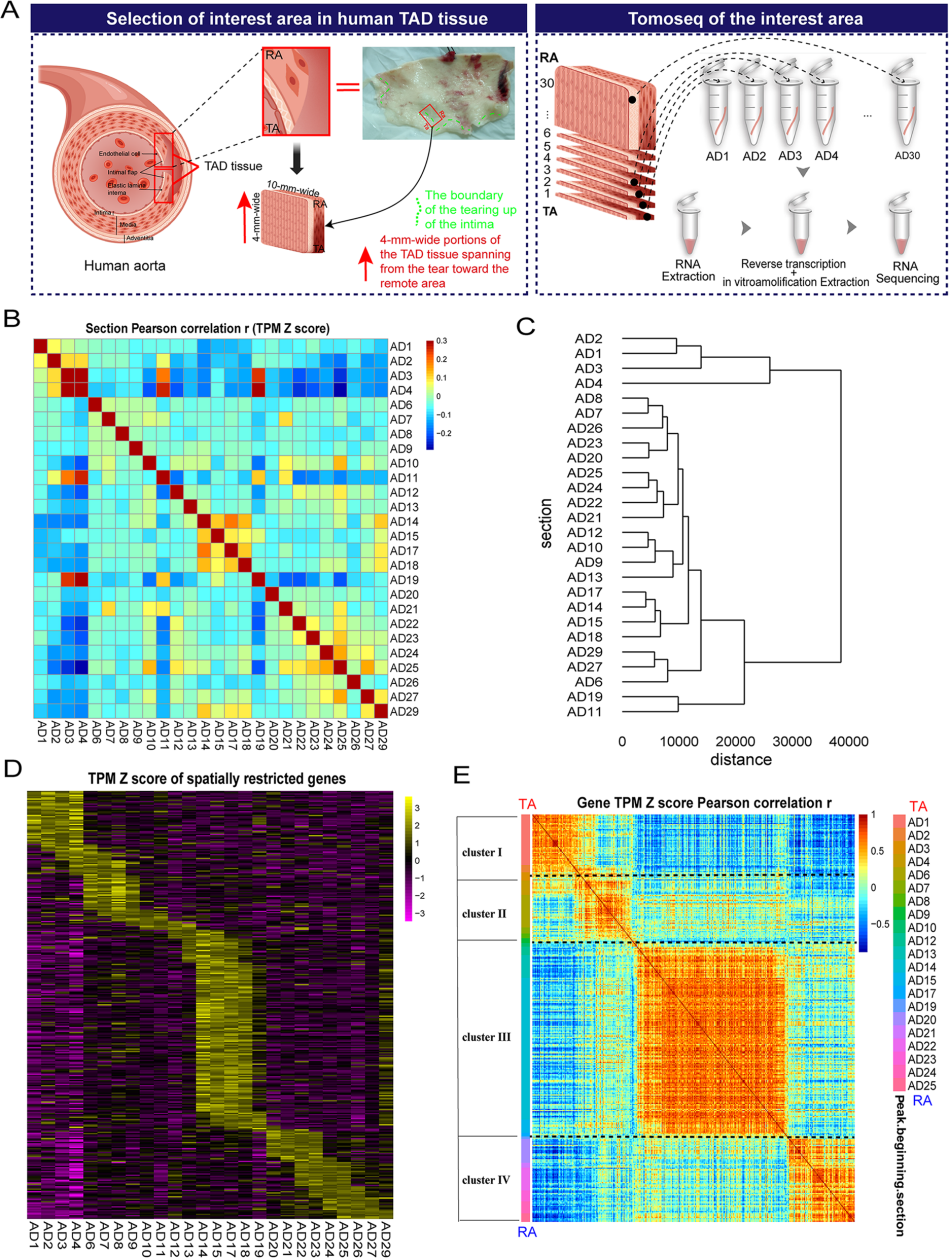
High-resolution gene expression atlas of the TAD tissue by Tomo-seq. **A** We obtained the TAD tissue and cut it perpendicular to the tear to approximately 4 mm wide and 1 cm long as the region of interest. And the schematic diagram of Tomo-seq. **B** Heatmap showing the pairwise correlation of gene expression profiles between sections. Red indicated a high correlation, and blue indicated a low correlation. **C** Dendrogram showing the hierarchical clustering of sections by gene expression profiles. Sections AD1 ~ AD4 showed a clear separation from other sections. **D** Heatmap showing the spatially restricted expression of genes that were highly expressed in at least 4 consecutive sections. Rows were genes, and columns were sections. Yellow indicates high expression, and purple indicated low expression. **E** Heatmap showing the pairwise correlation of expression pattern in sections between identified locally expressed genes. Red indicates a high correlation, and blue indicates a low correlation. The left bar shows the beginning of the high-expression peak among sections. Cluster I–IV indicates hierarchical clustering of expression traces for all genes that were found to be differentially expressed in **D**

Intimal flaps are communication sites between the true lumen and the false lumen [1] (Fig. 3A). To obtain precise spatial information molecular signatures, we applied Tomo-seq from TA to RA of intimal flaps. For this purpose, we obtained the frozen aorta (4.0 mm wide and 1.0 cm long) perpendicular to the tear boundary and processed it into ≈3000 µm continuous cryosections spanning from the TA to the RA (Fig. 3A). Additional file 1: Fig. S3A described the protocol for frozen tissue section tissue cryosectioning and how to optimize the frozen section parameters to obtain higher-quality RNA (Additional file 1: Fig. S3B). The results are shown in Additional file 3: Table S2–S4. Twenty consecutive sections with a thickness of 5μm were collected into an individual centrifuge tube. A total of 30 tubes were collected and labeled sequentially as AD1, AD2 up to AD30. RNA extracted from each tube was subsequently sequenced (Fig. 3A, Additional file 3: Table S5). The sections AD5, AD16, AD 28, and AD 30 had low RNA concentrations, which were excluded.

Genes expressed in at least 3 sections (TPM > 0) were selected for subsequent analysis, and a total of 26,417 genes were included (Additional file 3: Table S6, Additional file 4). We performed Pearson’s correlation analysis on the Z score of all genes for each pairwise combination of sections. We found three significant boundaries, including AD4, AD11, and AD19, suggesting a spatial division of gene expression (Fig. 3B). We next performed a hierarchical clustering analysis to analyze the correlation of gene expression in contiguous sections. The dendrogram showed at least 4 zones with specific molecular profiles from TA to RA, among which the highest correlation for AD1-AD4 and showed a clear separation from other sections (Fig. 3C). To further identify such regions, we followed the criteria (*Z* score > 1 in ≥ 4 consecutive sections, could identify highly reliable locally expressed genes) used by Wu et al. [11]. The ranked lists of 339 spatially upregulated genes were generated (Additional file 5). In addition, we used permutation tests to determine the statistical significance of locally expressed genes. *Z* score > 1 in ≥ 4 consecutive sections guarantees that 337 out of 339 genes had adjusted *p*-values < 0.05. The spatial separation of gene expression patterns became significantly more pronounced (Fig. 3D). The heatmap confirmed that these 339 locally expressed genes were roughly subdivided into 4 distinct clusters (Fig. 3E). AD1–AD25 indicated the section number starting with genes that show a clear expression peak (*Z* score > 1 in ≥ 4 consecutive sections) in the local area. Cluster I (AD1-AD2) means ~ 48 genes with a clear expression peak in the section AD1 ~ AD4 or AD2 ~ AD6 range. Cluster II (AD3-AD10) means ~ 62 genes with a clear expression peak in the section AD3 ~ AD7 or AD4 ~ AD8 or AD6 ~ AD9 or AD7 ~ AD10 or AD8 ~ AD11 or AD9 ~ AD12 or AD10 ~ AD13 range. Cluster III (AD12-AD19) means ~ 160 genes with a clear expression peak in the section AD12 ~ AD15 or AD13 ~ AD17 or AD14 ~ AD18 or AD15 ~ AD19 or AD17 ~ AD20 or AD18 ~ AD21 or AD19 ~ AD22 range. Cluster IV (AD20-AD25) means ~ 69 genes with a clear expression peak in the section AD20 ~ AD23 or AD21 ~ AD24 or AD22 ~ AD25 or AD23 ~ AD26 or AD24 ~ AD27, AD25 ~ AD29 range (Fig. 3E, Additional file 5). Dimensionality reduction of Z values of these locally expressed genes using t-SNE showed a similar result (Additional file 1: Fig. S4).

### Tomo-seq revealed localized remodeling responses within the dissection aorta

**Fig. 4:**
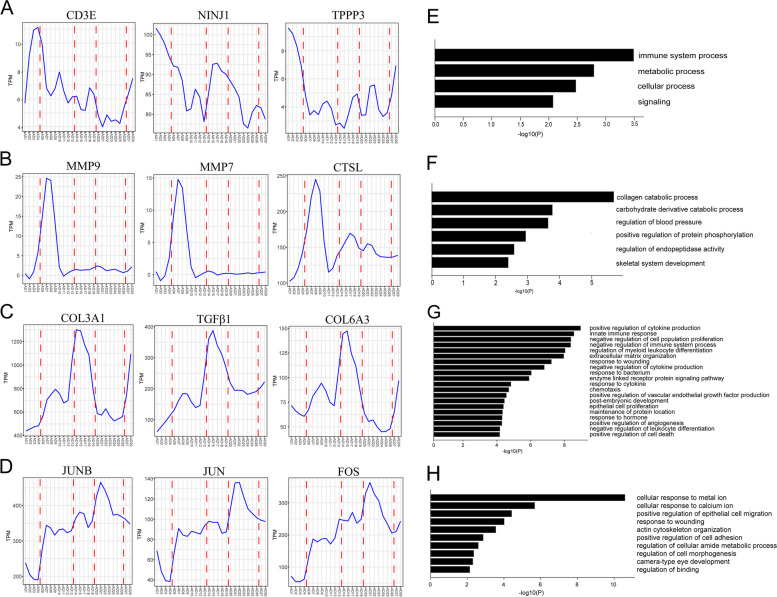
Spatial expression pattern of genes from the TA to the RA of the TAD tissue. **A** Spatial expression pattern of genes in cluster I, such as CD3, NINJ1, and TPPP3. **B** Spatial expression pattern of genes in cluster II, such as MMP9, MMP7, and CTSL. **C** Spatial expression pattern of genes in cluster III, such as COL3A1, TGFβ1, and COL6A3. **D** Spatial expression pattern of genes in cluster IV, such as JUNB, JUN, and FOS. **E** Results of GO-term analysis using metascape for genes with spatially restricted expression in cluster I. **F** Results of GO-term analysis using metascape for genes with spatially restricted expression in cluster II. **G** Results of GO-term analysis using metascape for genes with spatially restricted expression in cluster III. **H** Results of GO-term analysis using metascape for genes with spatially restricted expression in cluster IV

We further analyze the locally expressed genes in detail. We visualized the trajectories of these gene expression patterns using line plots (Fig. 4).

T cell marker gene, CD3E, was more abundantly expressed in cluster I. The gene in cluster I also contained genes associated with immune infiltrates like NINJ1 and TPPP3 (Fig. 4A). The matrix metalloproteinases (MMPs), specifically MMP9 and MMP7, were found to be more abundantly expressed near the TA. Furthermore, cathepsin L (CTSL), a lysosomal protease, exhibited a similar expression pattern and also clustered within Cluster II (Fig. 4B). MMPs and cathepsin were crucial in extracellular matrix degradation and elastin degradation [24, 25]. Collagens, including COL3A1 and COL6A3, were found to be more abundantly expressed in the region between TA and RA (Cluster III). Notably, TGFβ1 displayed similar expression patterns in this area (Fig. 4C). Overactivation of TGF-β signaling and collagen deposition were typical features in TAD [26]. JUN and FOS, which could form a dimeric transcription factor called AP-1, showed more abundantly expressed in RA (Cluster IV) (Fig. 4D). The upregulation of AP-1 signaling has been reported to have a beneficial effect on the prevention of TAD, including reducing monocyte graft infiltration and maintaining the integrity of elastin [27].

GO-term analysis revealed that genes in cluster I were associated with the “immune system process,” “metabolic process,” and “cellular process.” (Fig. 4E). Genes in cluster II were associated with the “collagen catabolic process,” “regulation of blood pressure,” and “skeletal system development.” (Fig. 4F). Interestingly, genes with expression peaks in cluster III were linked to “positive regulation of cytokine production,” “innate immune response,” “extracellular matrix organization,” and “response to wounding,” (Fig. 4G). Genes in cluster IV were associated with “cellular response to the metal ion,” and “response to wounding” (Fig. 4H). The results above indicated that TAD exhibited distinctive remodeling features across TA to RA.

### Tomo-seq identified potential new players in the formation of TAD

**Fig. 5:**
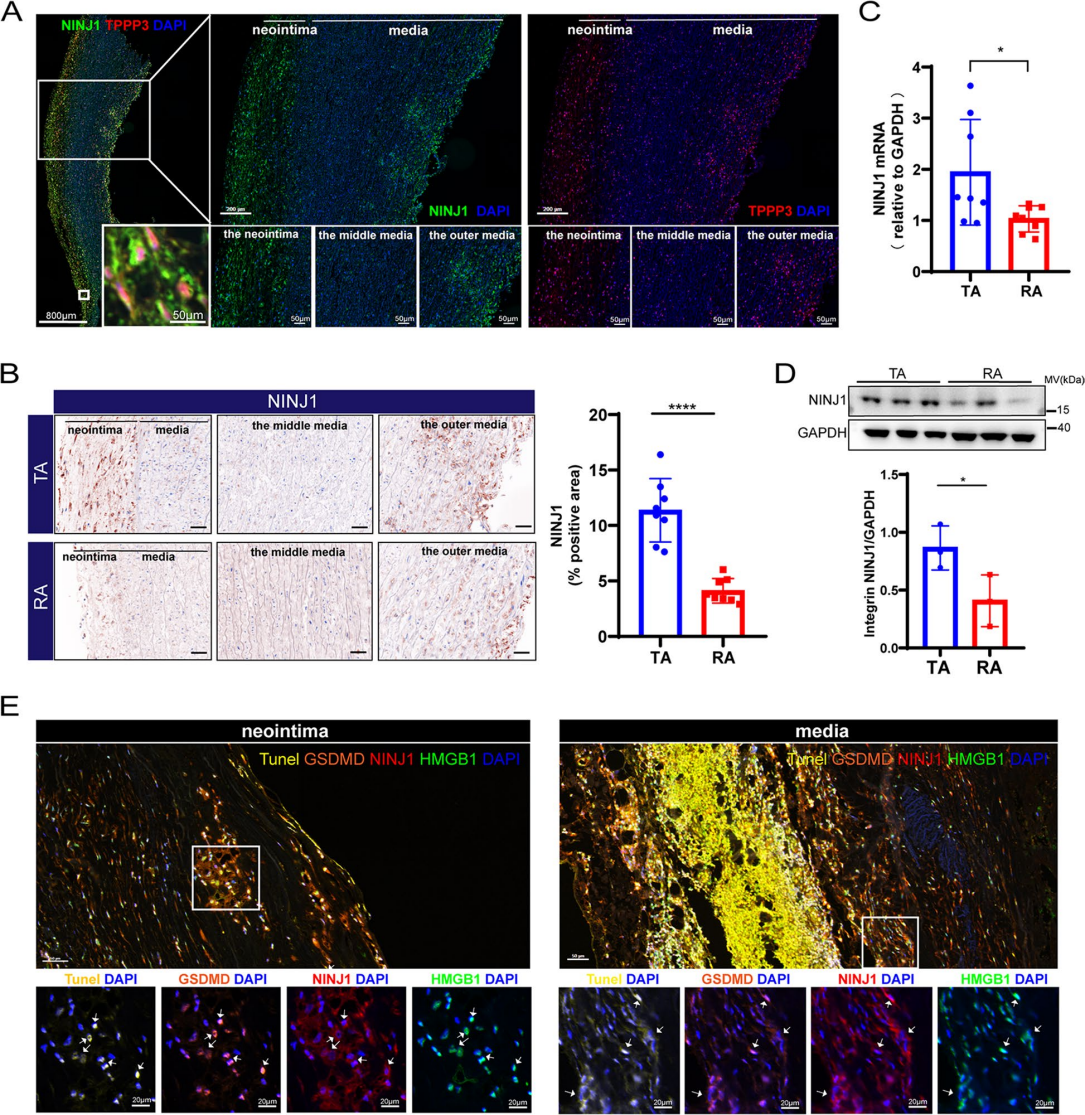
Validation of the trend of NINJ1 identified in cluster I. **A** Immunostaining for NINJ1 co-localized with TPPP3 in the human TAD tissue. **B** Representative images of immunostaining for NINJ1 in the TA and the RA, and quantification of the NINJ1 positive area (*n* = 8/group). Scale bar indicates 100 μm. **C** Validate high expression of NINJ1 in TA by qPCR (*n* = 8/group). **D** Validate high expression of NINJ1 in TA by WB. **E** Multilabel immunofluorescence showed NINJ1 was co-localized with cell death marker Tunel and GSDMD, and inflammation marker HMGB1, both in the neointima and media. *P < 0.05, **P < 0.01, ***P < 0.001, ****P < 0.0001; quantitative analysis of NINJ1 positive area was calculated as the ratio of positive area/total area of tissue section; quantitative data were shown as mean ± SEM; statistical analysis was performed with Student’s *t* test (**C**, **B**, **D**)

To validate local expression patterns of in the TA identified by Tomo-seq, we tested the expression of the top-ranked genes NINJ1 and TPPP3 in cluster I. Immunostaining showed that NINJ1 and TPPP3 were co-expressed in TAD tissues (Fig. 5A). NINJ1 was intensive in the neointima and the outer disorganized medial layers, but weak in the regular middle media (Fig. 5A). TPPP3 expression in TAD tissues showed the same expression pattern as NINJ1 (Fig. 5A). We further validated their local expression trends from the TA to the RA. The results confirmed that the positive area of NINJ1 in the TA was significantly higher than that in the RA, and NINJ1 was relatively lowly detectable in the RA (TA vs RA, 11.37 ± 2.51% vs 4.12 ± 0.97%, *p* < 0.0001, Fig. 5B). For TPPP3, we found that its positive area in the TA was significantly higher than the RA (Additional file 1: Fig. S5A). These results confirmed the spatial difference of NINJ1 and TPPP3 obtained by Tomo-seq.

To further validate the gene correlation of NINJ1 in TA, we examined genes which were in proximity with NINJ1. CD3E and NINJ1(upregulated in cluster I) had an expression peak within AD1–AD4, and MMP9 (upregulated in cluster II) also had an expression peak near the TA. Activation of T cells in AD could exacerbate disease progression by producing cytokines and MMPs and promoting aortic inflammation and destruction [28]. Other studies also demonstrated that MMP-9 showed increased expression in the TA [6]. Multiple labeling staining showed that CD3, NINJ1, and MMP9 were co-expressed and abundantly aggregated in TAD tissues (Additional file 1: Fig. S5B). The quantification of CD3, NINJ1, and MMP9 showed higher expression in the TA than that in the RA (Additional file 1: Fig. S5C, D).

NINJ1 could mediate plasma membrane rupture during lytic cell death [13]. NINJ1 also could mediate tissue remodeling and inflammation by regulating leukocyte entry, adhesion, activation, and movement [15]. Cell death and inflammation are inextricably linked to the development of aortic remodeling [29]. Our results also showed that the TA region had more apoptotic cells and immune cells. A recent study showed that inhibiting membrane rupture with NINJ1 antibodies could limit tissue injury [30]. The role of NINJ1 in TAD had not been illustrated. We further characterized the expression of NINJ1 in TA and RA by qPCR and WB, and the results suggested that NINJ1 was highly expressed in TA both at the transcriptome (*p* = 0.0292) (Fig. 5C) and protein level (*p* = 0.0462) (Fig. 5D, Additional file 1: Fig. S6). Multilabel immunofluorescence showed NINJ1 was co-localized with cell death marker such as GSDMD and Tunel, and proinflammatory marker HMGB1, both in the neointima and the outer disorganized medial layers (Fig. 5E). We consequently proposed the novel hypothesis that NINJ1 may be a new player in the formation of TAD.

**Fig. 6:**
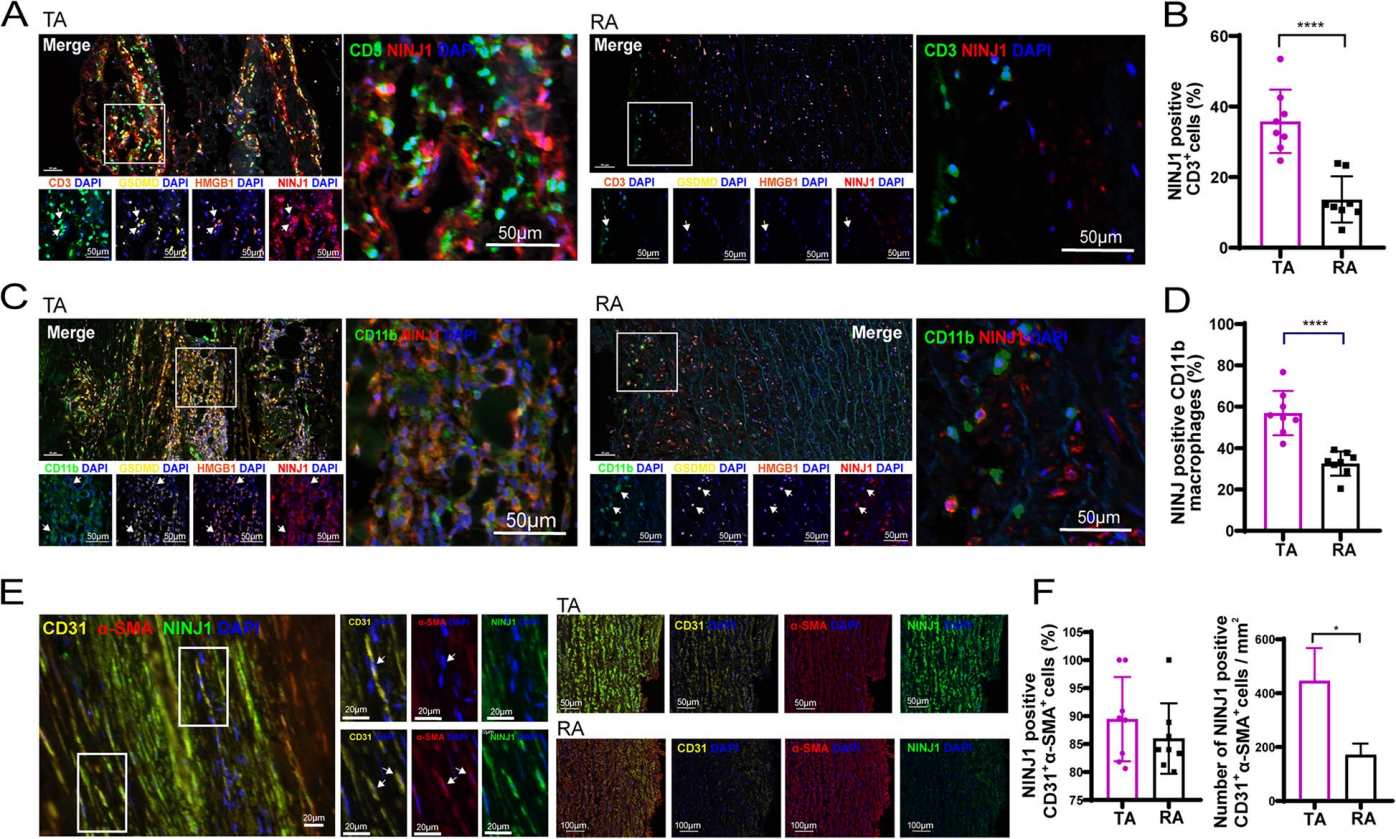
Multiple immunofluorescence detection on the NINJ1-expressing cells. **A** Immunofluorescent staining of CD3, GSDMD, HMGB1, and NINJ1 in TA and RA. **B** The quantitative analysis of the expression proportion of NINJ1 in CD3^+^ T cells between TA and RA. **C** Immunofluorescent staining of CD11b, GSDMD, HMGB1, and NINJ1 in TA and RA. **D** The quantitative analysis of the expression proportion of NINJ1 in CD11b^+^ macrophages between TA and RA. **E** Immunofluorescent staining of NINJ1, CD31, and α-SMA in TA and RA. **F** The quantitative analysis of the number of NINJ1^+^CD31^+^α-SMA^+^ cells per mm^2^ between TA and RA. In B, D and F, *n* = 8 for each group; *P < 0.05, **P < 0.01, ***P < 0.001, ****P < 0.0001; quantitative analysis of NINJ1 positive cells was calculated as the ratio of the number of NINJ1 positive points in these cells /a total number of CD3^+^/CD11b^+^/ CD31^+^α-SMA.^+^ cells in the tissue section; quantitative data were shown as mean ± SEM; Statistical analysis was performed with Student’s *t* test (**B**, **D**, **F**)

We next performed multilabel immunofluorescence to identify which types of cells highly expressed NINJ1 in the aorta of TAD. The results suggested that NINJ1 was predominantly expressed in macrophages, T cells, and CD31^+^α-SMA^+^ double-positive cells. About 35.83 ± 9.02% of CD3^+^ T cells in the TA expressed NINJ1, while only 13.67 ± 6.57% of T cells in the RA expressed NINJ1 (*p* < 0.0001, Fig. 6A, B). 56.98 ± 9.98% of CD11b^+^ macrophages in the TA area expressed NINJ1, only 32.61 ± 5.89% of macrophages in the RA area expressed NINJ1 (*p* < 0.0001, Fig. 6C, D). These NINJ1-positive immune cells also co-localized with GSDMD and HMGB1. NINJ1 was expressed at a higher ratio in CD31^+^ α-SMA^+^ double positive cells, regardless of TA or RA. However, TA contained more NINJ1^+^ CD31^+^ α-SMA^+^ cells (TA vs RA, 412.38 ± 121.87 vs 171.18 ± 42.09 per mm^2^, *p* = 0.001, Fig. 6E, F). The above results suggested that NINJ1 was associated with both inflammation, intima, and media remodeling in TAD.

### Knockdown of NINJ1 attenuated TAD formation

**Fig. 7:**
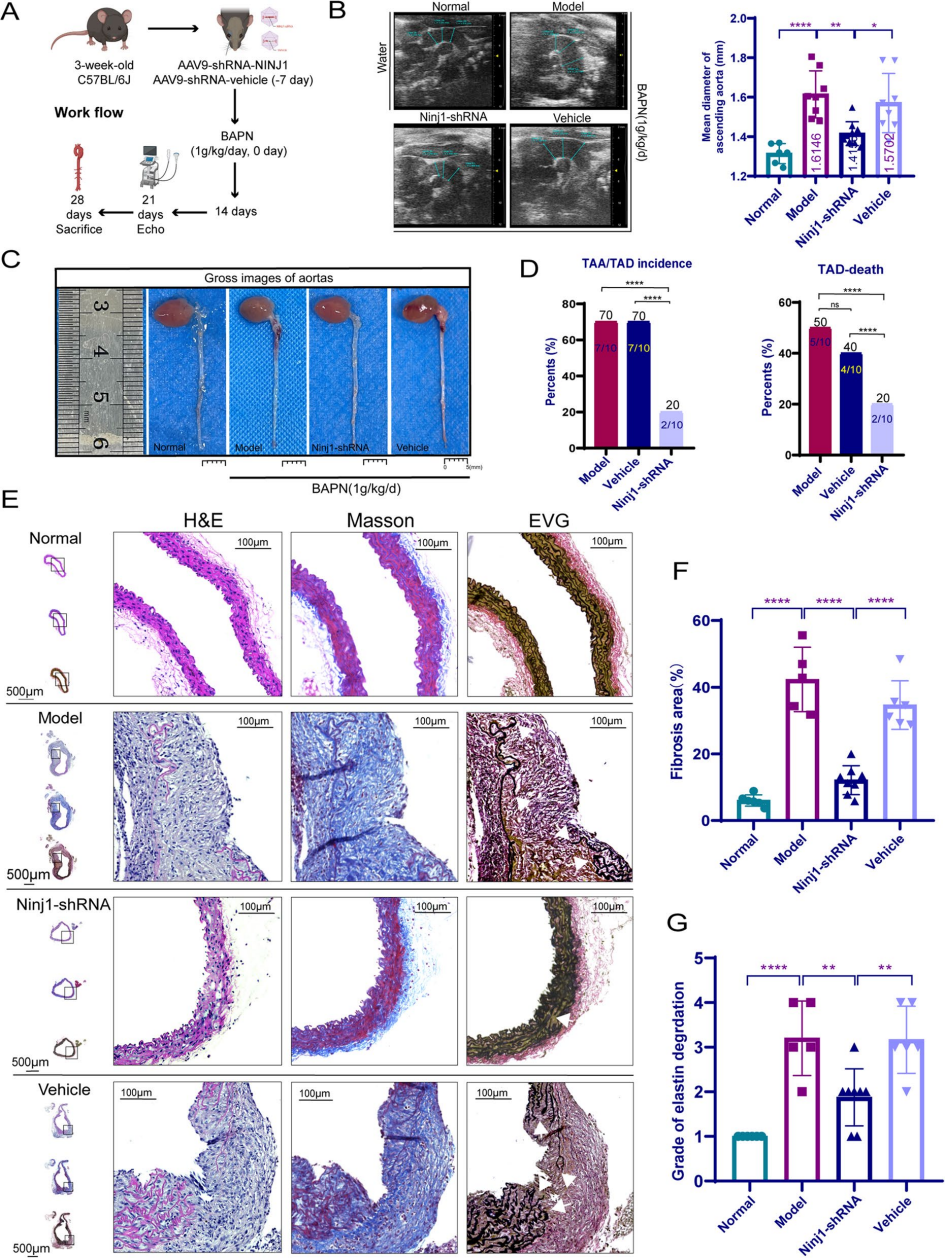
Knockdown of NINJ1 attenuated TAD formation. **A** Workflow for in vivo AAV9-Ninj1-shRNA knockdown experiments. **B** Ultrasonographic examination of the thoracic aorta mean diameter in each group of surviving mice after 3 weeks (*n* = 6, 8, 9, and 8, respectively). **C** Representative photographs of aortas in each group. **D** The incidence and mortality of TAA/TAD in each group at the end time of the experiment. **E** Representative H&E, Masson, and EVG staining of the mouse thoracic aortas in each group. Arrows indicate the elastin disruption. **F** Quantitative analysis of thoracic aortic fibrosis area in each group (*n* = 6, 5, 8, and 6, respectively). **G** Grade of the elastin degradation in the aortic wall (*n* = 6, 5, 8, and 6, respectively). *P < 0.05, **P < 0.01, ***P < 0.001, ****P < 0.0001; quantitative data were shown as mean ± SEM; statistical analysis in **B**, **F**, and **G** was performed with one-way ANOVA with Tukey’s tests, and chi-square test in **D**, respectively

To further demonstrate the relationship between NINJ1 and TAD. In this study, 3-week-old male C57BL/6J mice were injected with adeno-associated virus (AAV) 9-shRNA-Ninj1 or AAV9-shRNA-vehicle via post-glomus venous, respectively (Fig. 7A). The expression of NINJ1 could be decreased to 50.6% of normal by AAV9-shRNA-Ninj1 after 4 weeks (4.25 ± 0.60 vs 2.10 ± 0.25, *p* = 0.0003, Additional file 1: Fig. S7A, B). 7 days after the AAV9 injection, we began to construct the BAPN-induced TAD mice model, as shown in the flow chart in Fig. 7A. On day 21 after constructing the model, we performed ultrasonographic examination of the thoracic aorta diameter in each group. The results suggested that the Ninj1-shRNA substantially reduced the mean diameter of the thoracic aorta (1.42 ± 0.07 mm) compared with that in model mice (1.61 ± 0.11 mm, *p* = 0.0031) and vehicle mice (1.57 ± 0.14 mm, *p* = 0.0263) (Fig. 7B). On day 28 after constructing the model, the mice in each group were sacrificed. Gross pathology showed that Ninj1-shRNA significantly reduced the incidence of TAA/TAD (20%) and mortality (20%) compared with those in model mice (incidence of 70%, mortality of 50%, respectively, both *p* < 0.0001) and vehicle mice (incidence of 70%, mortality of 40%, respectively, both *p* < 0.0001) (Fig. 7C, D). We further evaluated the pathological changes by H&E, Masson, and EVG staining, respectively (Fig. 7E). The results suggested that Ninj1-shRNA significantly reduced the level of fibrosis (Model vs Ninj1-shRNA vs Vehicle, 42.34 ± 8.62% vs 12.14 ± 4.07% vs 34.70 ± 6.67%, *p* < 0.0001, Fig. 7F) and elastic fiber disruption and degeneration (*p* < 0.01, Fig. 7G) of the aortic wall in the TAD model. The above suggested that NINJ1 was closely associated with the formation of TAD.

Ninj1-shRNA significantly decreased both the infiltration of T cells (Model vs Ninj1-shRNA vs Vehicle, 1142.97 ± 304.66 vs 348.58 ± 65.44 vs 841.32 ± 133.60 per mm^2^, *p* < 0.0001 and *p* = 0.0073) and macrophages (Model vs Ninj1-shRNA vs Vehicle, 1096.63 ± 286.34 vs 249.63 ± 51.54 vs 810.64 ± 203.18 per mm^2^, *p* < 0.0001 and *p* = 0.0006) within the aorta compared with that in model mice and vehicle mice (Additional file 1: Fig. S7C, D), suggesting that NINJ1 was associated with the inflammation during the formation of TAD. Meanwhile, we also observed the co-expression of NINJ1, CD31, and α-SMA in the aorta of the TAD model (Additional file 1: Fig. S7E). Furthermore, we found that the CD31 signaling exhibited greater integrity within the endothelial monolayer of the Normal and Ninj1-shRNA groups. Conversely, in the model and vehicle groups, α-SMA signaling demonstrated significant proliferation and expression within the endothelial layer, accompanied by reduced CD31 signaling in the endothelial monolayer and notable proliferation in the medial layers (Additional file 1: Fig. S7F). The number of CD31^+^α-SMA^+^ cells was significantly decreased by Ninj1-shRNA (Additional file 1: Fig. S7F). Tunel^+^ cells were also diminished by Ninj1-shRNA as observed in Additional file 1: Fig. S7G, H. The above suggested that inhibition of NINJ1 may have a role in suppressing inflammation infiltration, cell death, and EndMT-related vascular remodeling in TAD.

### NINJ1-neutralization antibody limited TAD formation

**Fig. 8:**
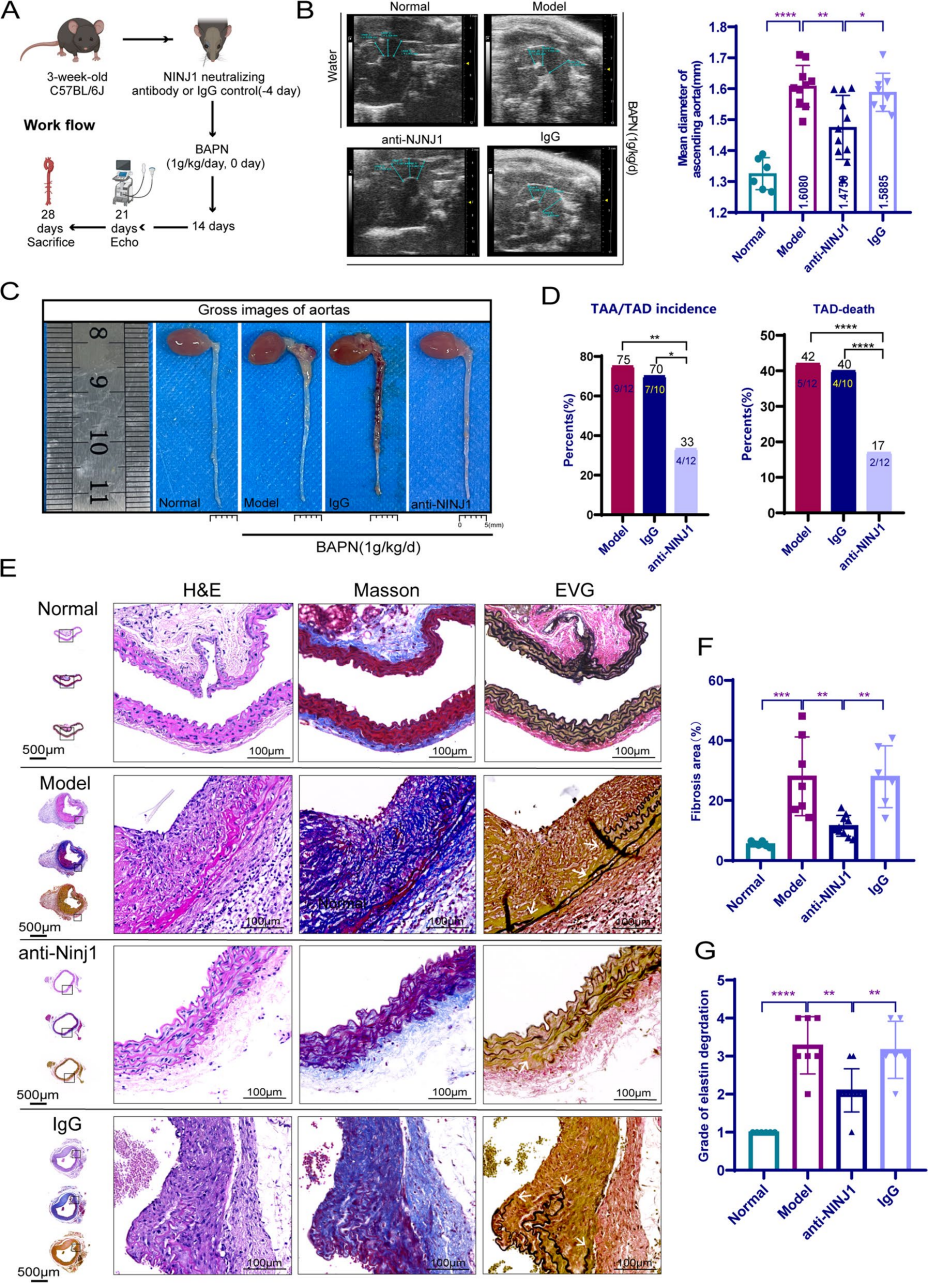
NINJ1-neutralization antibody limited TAD formation. **A** Workflow for NINJ1-neutralization antibody treatment experiments. **B** Ultrasonographic examination of the thoracic aorta mean diameter in each group of surviving mice after 3 weeks (*n* = 6, 10, 11, and 8, respectively). **C** Representative photographs of aortas in each group. **D** The incidence and mortality of TAD in each group at the end time of the experiment. **E** Representative H&E, Masson, and EVG staining of the mouse thoracic aortas in each group. Arrows indicate the elastin disruption. **F** Quantitative analysis of thoracic aortic fibrosis area in each group (*n* = 6, 7, 10, and 6, respectively). **G** Grade of the elastin degradation in the aortic wall (*n* = 6, 7, 10, and 6, respectively). *P < 0.05, **P < 0.01, ***P < 0.001, ****P < 0.0001; quantitative data were shown as mean ± SEM; statistical analysis in **B**, **F**, and **G** was performed with one-way ANOVA with Tukey’s tests, and chi-square test in **D**, respectively

To investigate the potential therapeutic effect of NINJ1-neutralizing antibody on TAD. In this study, 3-week-old male C57BL/6J mice were pretreated with a single shot of NINJ1-neutralizing antibody (*n* = 12) or control IgG antibody (*n* = 10) via post-glomus venous injection. Four days after the pretreatment, we constructed the BAPN-induced TAA/TAD mice model, as depicted in the flow chart shown in Fig. 8A. On day 21 after constructing the model, we conducted an ultrasonographic examination of the thoracic aorta diameter in each group. The results indicated that the NINJ1-neutralizing antibody significantly reduced the mean diameter of the thoracic aorta (1.48 ± 0.10 mm) compared with that in model mice (1.61 ± 0.06 mm, *p* = 0.0025) and mice treated with IgG (1.59 ± 0.06 mm, *p* = 0.0183) (Fig. 8B). On day 28 after constructing the TAD model, the mice in each group were sacrificed. The results demonstrated that inhibition of NINJ1 significantly reduced the incidence of TAA/TAD (33%) and mortality (17%) compared with those in model mice (incidence of 75%, mortality of 42%, respectively, *p* = 0.0047 and *p* < 0.0001) and treated with IgG (incidence of 70%, mortality of 40%, respectively, *p* = 0.012 and *p* < 0.0001) (Fig. 8C, D). We further evaluated the pathological changes using H&E, Masson, and EVG staining, respectively (Fig. 8E). The results suggested that the NINJ1-neutralizing antibody significantly reduced the level of fibrosis (Model vs Anti-NINJ1 antibody vs IgG, 28.05 ± 12.13% vs 11.54 ± 3.29% vs 27.93 ± 9.40%, *p* = 0.0021 and *p* = 0.0035, Fig. 8F) and elastic fiber disruption and degeneration of the aortic wall (*p* < 0.01, Fig. 8G) in the TAD model. Overall, these findings indicate that the NINJ1-neutralizing antibody had a therapeutic effect on TAD.

The TAD model exhibited a notable decrease in NINJ1 expression following treatment with NINJ1-neutralizing antibody (Additional file 1: Fig. S8A, B). Additionally, the presence of T cells (Model vs Anti-NINJ1 antibody vs IgG, 1061.94 ± 199.66 vs 386.62 ± 83.91 vs 923.03 ± 136.51 per mm^2^, both *p* < 0.0001) and macrophages (model vs Anti-NINJ1 antibody vs IgG, 1352.90 ± 363.26 vs 291.74 ± 50.96 vs 1170.86 ± 312.71 per mm^2^, both *p* < 0.0001) within the aorta was significantly diminished upon administration of NINJ1-neutralizing antibody compared to model mice and mice treated with IgG (Additional file 1: Fig. S8A, B). The administration of NINJ1 neutralizing antibody resulted in a reduction in the population of CD31^+^α-SMA^+^ cells (Additional file 1: Fig. S8C). Tunel^+^ cells were also diminished by NINJ1-neutralizing antibody as observed in Additional file 1: Fig. S8D, E. These results indicated that NINJ1-neutralization antibody limited inflammation, tissue remodeling, and cell death.

### Small molecule drugs limited TAD formation

The above results showed that inhibition of NINJ1 may be a potential strategy to limit the development of TAD. Phenyl-β-D-glucopyranoside (PDG) is a recently isolated small molecule component of *Phellodendri amurensis*, a traditional medicine for anti-inflammatory, which has been shown to attenuate NINJ1 expression and downregulate MMP activity under lipopolysaccharide-stimulated inflammatory conditions [31, 32]. Thus, we also used PDG to treat the mice TAA/TAD model (Additional file 1: Fig. S9A). After 3 weeks, PDG significantly reduced the severity of TAA/TAD, including reducing fibrosis, the fracture of elastic fiber fractures, and the infiltration of T cells and macrophages (Additional file 1: Fig. S9B–H).

## Discussion

Our study has demonstrated the following: (1) Significant neointima formation, tissue fibrosis, media degeneration, and inflammatory infiltration were observed in the TA. (2) Tomo-seq analysis revealed a spatial partitioning in gene expression between the TA and the RA. Furthermore, it identified NINJ1 as a key molecule involved in inflammation and tissue remodeling of TAD. (3) In the human aorta, NINJ1 co-localized with macrophages, T cells, and CD31^+^α-SMA^+^ cells, primarily showing clustering at the TA. (4) Inhibition of NINJ1 significantly reduced the morbidity and mortality associated with BAPN-induced TAD in mice. Previous studies found lesions of TAD are regional and transmural specific, with the outer media of the ascending aorta being a susceptible site [26]. The present study demonstrated that neointima formation accompanied by inflammatory infiltration and fibrosis was also an essential feature of TAD, especially in the TA.

We utilized Tomo-seq to capture the continuous change trajectory of genes in human TAD tissue, spanning from the TA to the RA, and accurately identified genes associated with TAD remodeling. Following the Tomo-seq protocol [12, 16, 33], we employed a single-cryosection thickness of ≤ 10 μm and consecutive mixed slices to analyze specific localized information for sequencing. GO-term analysis of the genes expressed locally emphasized the typical steps in the complex process of TAD: inflammation and extracellular matrix organization [34]. Searching for genes that exhibit similar transcription patterns to these classic markers holds the promise of discovering novel key genes closely associated with TAD. Cluster I was at the edge of the TA and contained the well-known T cell marker CD3E and was enriched in the immune system process. T-cell activation plays a vital role in neointima formation in response to arterial injury [35]. Some studies have reached the conclusion that Th1 immune responses are positively correlated with vascular remodeling and intimal expansion of TAD [35, 36]. Our data showed novel genes associated with intimal inflammation in cluster I, including NINJ1 and TPPP3. Recent studies have shown that NINJ1 regulated the tissue remodeling and inflammation in vascular [15, 37]. Immunostaining analyses demonstrated that the co-location of NINJ1 with T cells and macrophages trended toward aggregation at the TA in human tissue. TPPP3, a member of tubulin polymerization-promoting proteins, which was involved in palmitic acid-induced endothelial oxidative injury [38, 39]. We verified that TPPP3 was also abundantly enriched in the neointima of TA.

NINJ1 was involved in multiple diseases and played various roles. Ninj1 could stimulate the inflammatory response of macrophages and thus promote the development of pulmonary fibrosis [40]. Leukocyte upregulation of Ninj1 could increase cell adhesion and transport in the inflammation of the central nervous system [37, 41]. NINJ1 was involved in endothelial dysfunction and inhibition of NINJ1 expression was a potential therapeutic strategy to prevent endothelial dysfunction in diabetes [42]. NINJ1 also could mediate plasma membrane rupture during lytic cell death [13]. The role of NINJ1 in TAD has not been explored. In our study, we observed that NINJ1 was enriched at the TA of TAD and co-localized with cell death markers, such as GSDMD and Tunel, and inflammation marker HMGB1. Therefore, we hypothesized that NINJ1 is closely associated with TAD.

We then utilized shRNA to downregulate NINJ1 expression in the BAPN-induced TAD model, which resulted in a notable reduction in TAD formation. Moreover, this approach also led to diminished infiltration of inflammatory cells and a decrease in the number of CD31^+^α-SMA^+^ cells. The NINJ1-neutralizing antibody also demonstrated comparable therapeutic effects and can effectively impede the formation of TAD. Inflammation can induce the generation of EndMT, and EndMT can in turn exacerbate inflammation, forming a vicious cycle [23, 43]. Our results showed inhibition of NINJ1 could reduce inflammation and EndMT, which is the potentially effective approach for treating TAD. On the other hand, we used PDG to treat BAPN-induced TAD models and found that it has an effect of delaying the development of TAD.

Our study also suggested that Tomo-seq would be suitable for various cardiovascular diseases. For example, Studies have shown that plaques associated with acute coronary syndrome exhibit more significant longitudinal heterogeneity. Therefore, at every single point in time, various parts of the same plaque may show different stages and trajectories [44, 45]. Tomo-seq can potentially facilitate the analysis of arterial remodeling mechanisms in plaque development. In valvular heart disease, continuous calcium deposition is the primary cause that leads to the gradual narrowing of the aortic valve [46]. Stenotic aortic valves existed in non-calcified regions and calcified regions [47]. Tomo-seq will help to understand the pathological progress of vascular diseases.

The limitation of this study is that we did not knock down NINJ1 in specific types of cells. The main reason for this is that we observed NINJ1 was expressed in multiple cell types associated with TAD progression, such as macrophages, T cells, and CD31^+^α-SMA^+^ cells. As a result, we chose to employ a broad NINJ1 inhibition to investigate the association between NINJ1 and TAD.

## Conclusions

For this study, we applied Tomo-seq to focus on the genes highly expressed in the TA and identified NINJ1 as a novel player in the inflammation of TAD. We demonstrated that NINJ1 was mainly enriched in the neointima and the media of the TA. Targeted NINJ1 may attenuate the development of TAD. We also emphasized that TAD lesions are regional and provided a new technical verification for studying the heterogeneity of vascular diseases.

### Supplementary Information

**Additional file 1:** **Fig. S1.** The schematic diagram of patient specimen collection. **Fig. S2.** Representative pictures of H&E staining of the TA and the RA of each patient. **Fig.S3.** Protocol for Tomo-seq on the human TAD tissue. **Fig. S4.** t-SNE plot of locally expressed genes. **Fig. S5.** Validation of the trend of gene expression similar to NINJ1 provided by Tomo-seq. **Fig. S6.** WB analysis for NINJ1 in TA and RA of human TAD tissue. **Fig. S7.** AAV9-Ninj1-shRNA limited inflammation, tissue remodeling, and cell death. **Fig. S8.** NINJ1-neutralization antibody limited inflammation, tissue remodeling, and cell death. **Fig. S9.** PDG inhibited BAPN-induced TAD formation.

**Additional file 2.** The ARRIVE guidelines 2.0: author checklist.

**Additional file 3:** **Table S1.** Main clinical characteristics of patients with TAD. **Table S2.** RNA concentration of different total slices thickness. **Table S3.** RNA concentration of ten consecutive sections with a thickness of 10µm. **Table S4.** RNA concentration of twenty consecutive sections with a thickness of 5µm. **Table S5.** Reads statistics results of TAD Cryosectioning. **Table S6.** Mapping of TAD Cryosectioning.

**Additional file 4.** Genes expressed in at least 3 sections (TPM＞0).

**Additional file 5.** The ranked lists of 339 spatially upregulated genes (Z score > 1 in ≥ 4 consecutive sections).

## Data Availability

Data are available on reasonable request. All data relevant to the study are. included in the article or uploaded as supplementary information. The datasets generated during the current study are available in the NCBI, https://dataview.ncbi.nlm.nih.gov/object/PRJNA811693?reviewer=csb73hf1rp4vtrh5tq3jen064c.

## Abbreviations

AAV: Adeno-associated virus
BAPN: Beta-aminopropionitrile
EndMT: Endothelial-to-mesenchymal transition
EVG: Verhoeff’s Van Gieson
GO-term: Gene ontology-term
H&E: Hematoxylin and eosin
MMP: Matrix metalloproteinases
NINJ1: Ninjurin 1
PDG: Phenyl-β-D-glucopyranoside
RA: Remote area
SEM: Standard error of the mean
shRNA: Short hairpin RNA
TA: Tear area
TAA: Thoracic aortic aneurysm
TAD: Thoracic aortic dissection
Tomo-seq: Tomography RNA sequencing
WB: Western blot
α-SMA: Alpha smooth muscle actin
