# Supplementary material for: Tomo-seq identifies NINJ1 as a potential target for anti-inflammatory strategy in thoracic aortic dissection
Source: BMC Med. 2023 Oct 20;21:396. doi: 10.1186/s12916-023-03077-1 (PMC10588060; doi:10.1186/s12916-023-03077-1)
Supplement: Supplementary file 1 — Additional file 1: Fig. S1. The schematic diagram of patient specimen collection. Fig. S2. Representative pictures of H&E staining of the TA and the RA of each patient. Fig.S3. Protocol for Tomo-seq on the human TAD tissue. Fig. S4. t-SNE plot of locally expressed genes. Fig. S5. Validation of the trend of gene expression similar to NINJ1 provided by Tomo-seq. Fig. S6. WB analysis for NINJ1 in TA and RA of human TAD tissue. Fig. S7. AAV9-Ninj1-shRNA limited inflammation, tissue remodeling, and cell death. Fig. S8. NINJ1-neutralization antibody limited inflammation, tissue remodeling, and cell death. Fig. S9. PDG inhibited BAPN-induced TAD formation. [file 12916_2023_3077_MOESM1_ESM.docx]

**Supplementary Information**

**Tomo-seq identifies NINJ1 as a potential target for anti-inflammatory strategy in thoracic aortic dissection**

**Authors names and affiliations:** Yixuan Sheng^1,5#^, Liying Wu^1,2#^, Yuan Chang^4, 5#^, Wendao Liu^5^, Menghao Tao^4, 5^, Xiao Chen^4,5^, Xiong Zhang^1^, Bin Li^1^, Ningning Zhang^4,5^, Dongting Ye^1^, Chunxi Zhang^1^, Daliang Zhu^1^, Haisen Zhao^1^, Aijun Chen^1^, Haisheng Chen^1*^ and Jiangping Song^3, 4, 5*^

^1^ Department of Cardiovascular Surgery, Guangzhou First People’s Hospital, School of Medicine, South China University of Technology, Guangzhou, China.

^2^ The First Clinical Medicine College of Guangzhou University of Chinese Medicine, Guangzhou, China.

^3^ Shenzhen Key Laboratory of Cardiovascular Disease, Fuwai Hospital Chinese Academy of Medical Sciences, Shenzhen 518057, China.

^4^ Beijing Key Laboratory of Preclinical Research and Evaluation for Cardiovascular Implant Materials, Animal Experimental Centre, Fuwai Hospital, National Centre for Cardiovascular Disease, Chinese Academy of Medical Sciences and Peking Union Medical College, Beijing 100037, China.

^5^ State Key Laboratory of Cardiovascular Disease, Department of Cardiac Surgery, National Centre for Cardiovascular Diseases, Fuwai Hospital, Chinese Academy of Medical Sciences and Peking Union Medical College, Beijing, China.

^#^ Contribute equally

* Corresponding authors

# Supplement figures


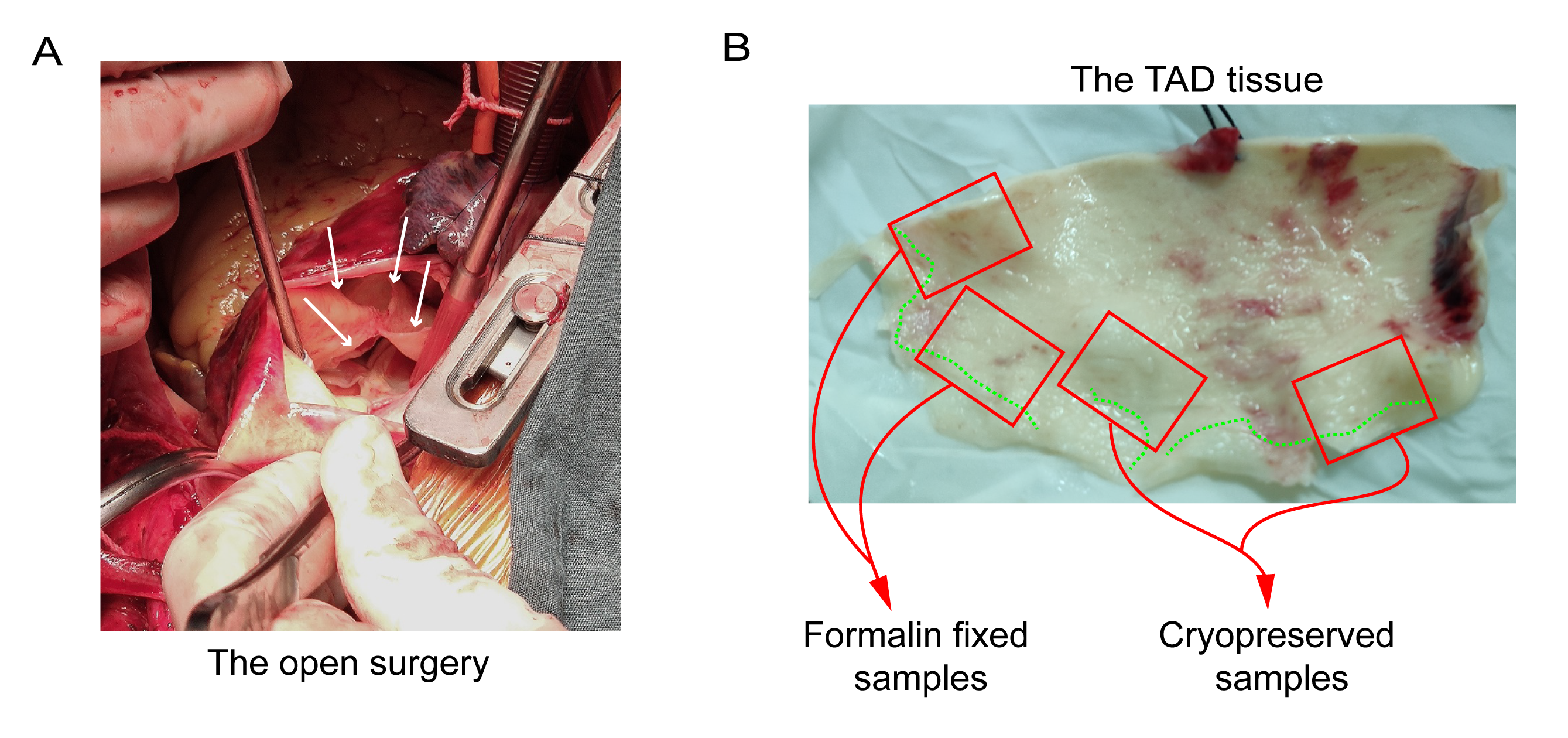


**Fig. S1. The schematic diagram of patient specimen collection**

**A** The TAD samples were obtained through open surgery, take pictures and record the boundary of the Tear. **B** All samples were collected within 30 minutes after the aorta excision and were repeatedly flushed with saline at 4°C to remove blood and mural thrombus adhering to the aortic wall, and the boundary of the Tear recorded by photographing. Afterwards, these samples were cut according to the needs of the study. One part containing the proximal tear lesions, was cut about 1cm in the direction perpendicular to the boundary of the Tear, keeping the distal edge and the boundary of the tear parallel to each other, place it in a 2ml eppendorf tube with the tear area downwards and store it in a refrigerator at -80°C until use. The remaining parts are cut in the same manner as reserve samples for frozen storage and fixed. The left panel visualizes the intraoperative sample, and white arrows indicated the location of aortic dissection Tears. The right panel showed the retention area of the sample. The green line segment was the boundary of the Tear, and the red boxs were the scope of the sample.


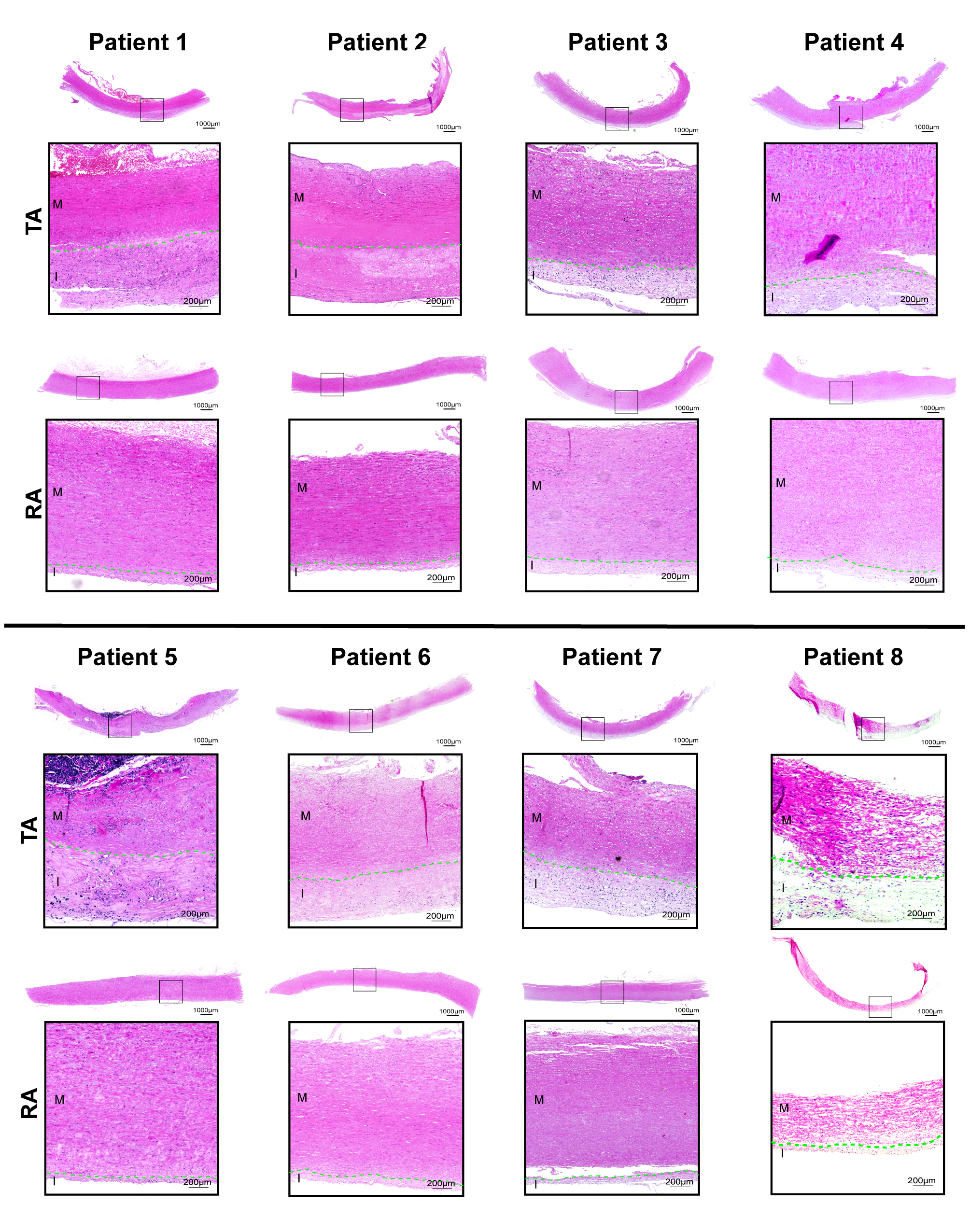


**Fig. S2. Representative pictures of H&E staining of the TA and the RA of each patient.**

The paired comparison of the pathological patterns in the TA and the RA. Severe neointimal hyperplasia was present in the TA, while no significant neointimal hyperplasia was observed in the RA. The green dotted line distinguishes between the neointima and the media. Scale bar, 1000 μm in the whole tissue section; 200 μm in the enlarged local image. TA, tear area; RA, remote area; **I** indicate the intimal layer; **M** indicates the media layer.


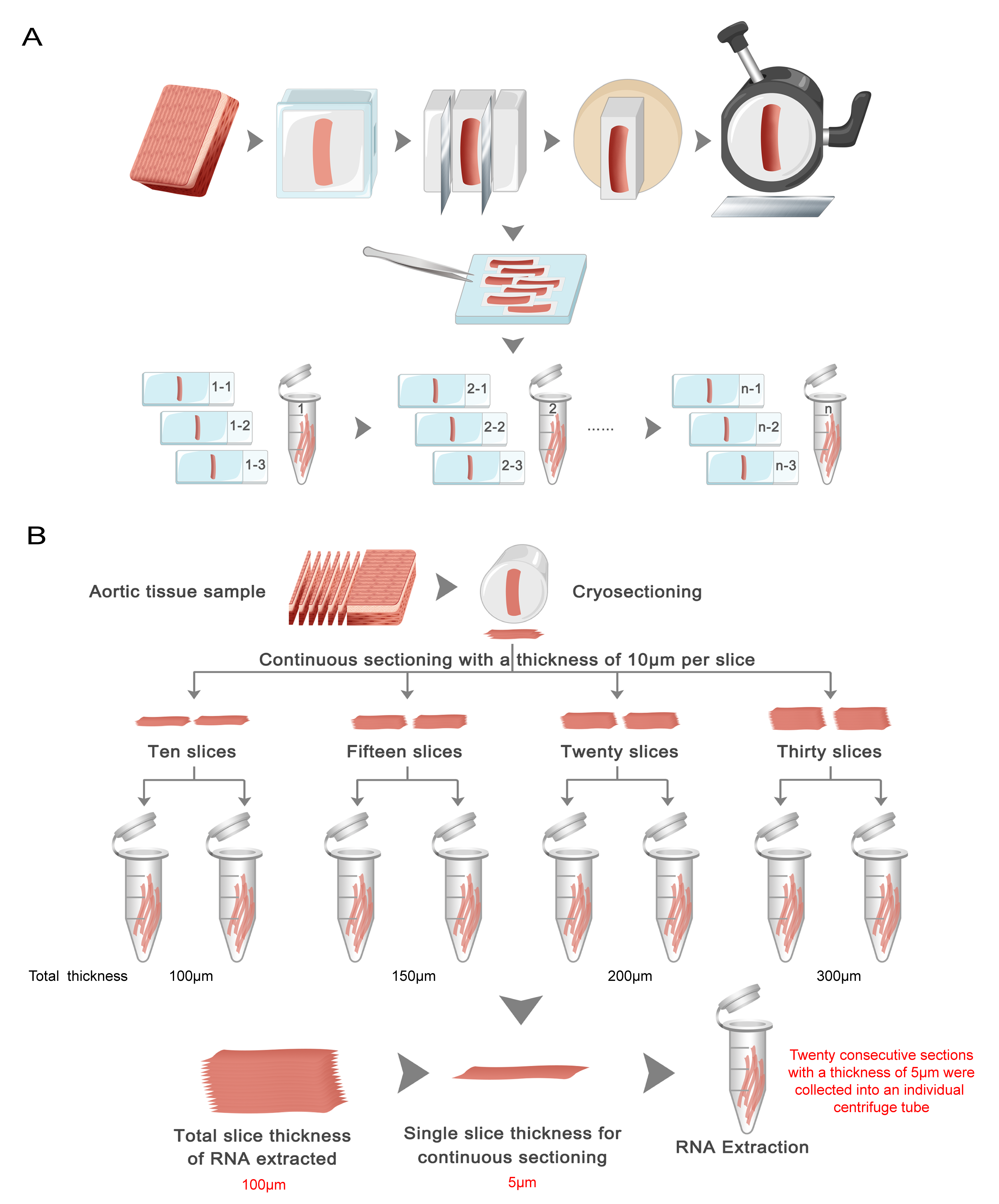


**Fig. S3. Protocol for Tomo-seq on the human TAD tissue.**

**A** **Protocol for Tissue Cryosectioning.** Take out the required samples from -80℃, as described in Fig. S1. The sample with a wide of about 1cm was reserved with a wide of about 4 mm for the Tomo-seq study. Then designed the slice direction according to the research purpose. Here, we obtained consecutive cryosections from the Tear area to the Remote. First, the tissue was embedded with OCT compound, When the OCT compound becomes extra-white, take it out from the embedding box, cut off the OCT compound without tissue as much as possible, and stick it to the freezer tray, Use the needle-nose tweezers to transfer the section to the centrifuge tube. Before each successive section, save three cryosections for subsequent staining analysis. **B Schematic diagram of parameter optimization for RNA Extraction.** We performed frozen section parameter optimization using aortic tissue from 3 patients. Initially, we collected several consecutive cryosectioning with a single section of 10µm thickness for RNA extraction to determine the total thickness of cryosections loaded into the single centrifuge tube. We collected consecutive 10 slices, 15 layers, 20 layers, and 30 slices, corresponding to a total thickness of 150 µm, 200 µm, and 300 µm, respectively, and made 2 replicates for each condition. Subsequently, we determined the total thickness of the section based on the RNA concentration of the extracted individual centrifuge tubes. Higher concentrations of RNA can be obtained with 10 consecutive layers and a total thickness of 100 µm. Then performed several replicate experiments to optimize the thickness of a monolayer section. We set the monolayer slices to 10 µm, and 5 µm, and collected 20 layers slices consecutively into one centrifuge tube, respectively, with 2 replicates per condition. Finally, twenty consecutive sections with a thickness of 5 µm were collected into an individual centrifuge tube as the optimal slicing parameters.


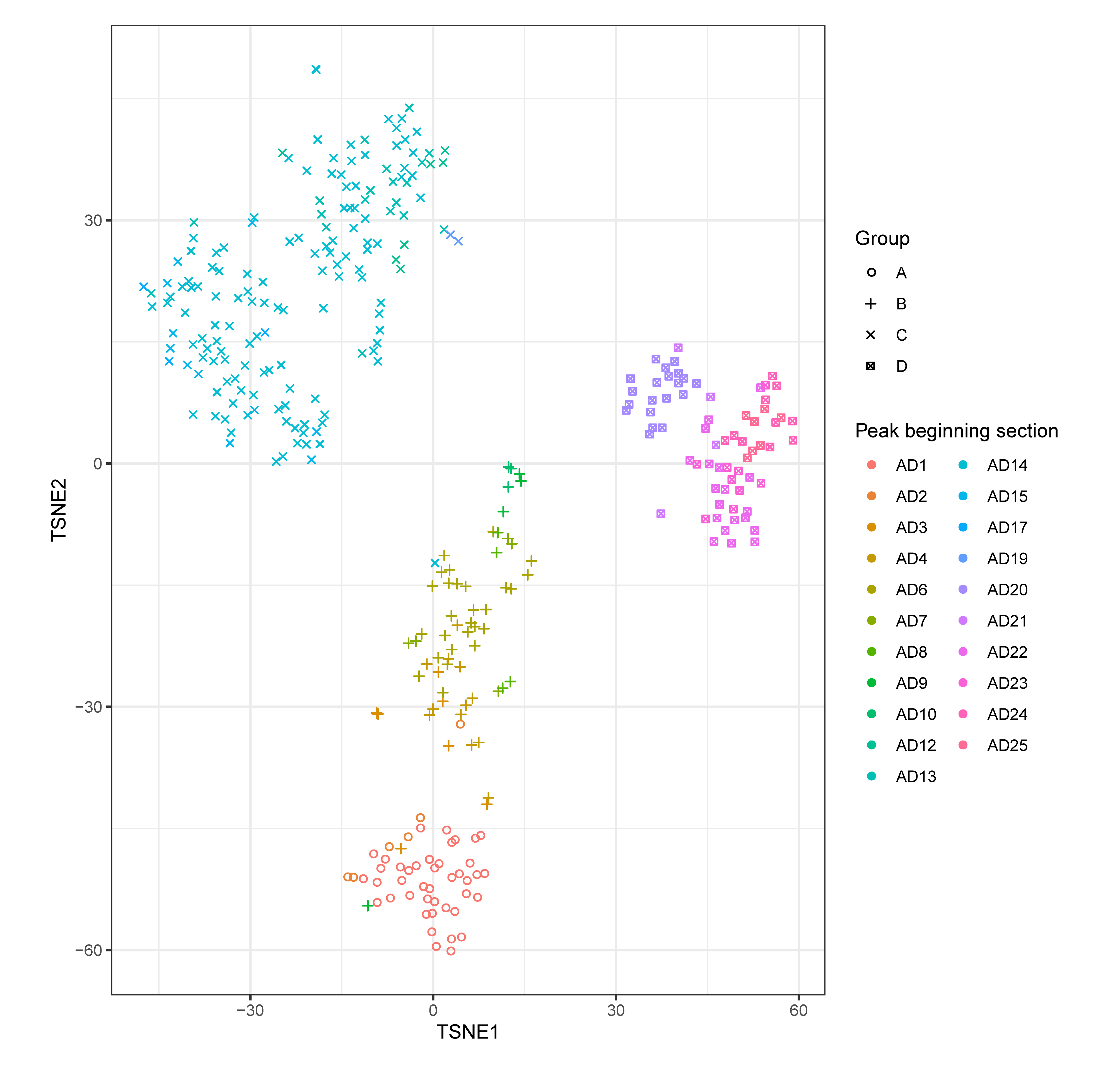


**Fig. S4. t-SNE plot of locally expressed genes**

Scatter plot showing grouped locally expressed genes according to expression pattern in sections. Each point corresponds to a gene. Shapes shows that there are four groups of genes with distinct expression pattern. Colors indicate the beginning section of spatially high expression (peak).


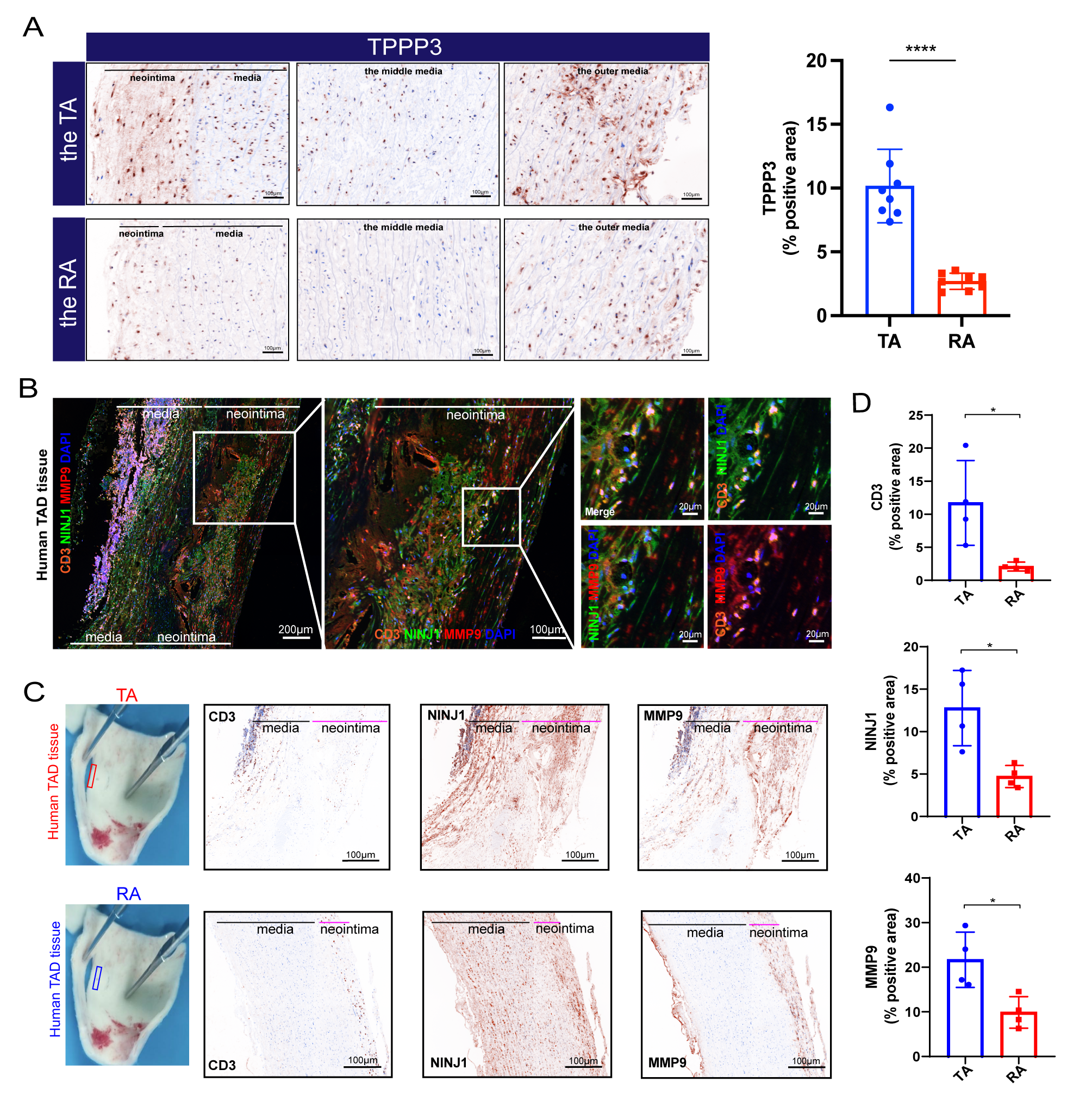


**Fig. S5.** **Validation of the trend of gene expression similar to NINJ1 provided by Tomo-seq**

**A** Representative image of immunostaining for TPPP3 in the TA and the RA, and quantification of the TPPP3 positive area. **B** Representative images of immunostaining for NINJ1 with co-staining for CD3 and MMP9 in the human TAD tissues. **C** Representative images of immunostaining for CD3, NINJ1, and MMP9 in the TA and the RA. **D** The quantitative analysis of NINJ1, CD3 and MMP9 in the TA and the RA. TA, tear area; RA, remote area. N=8 for each group in A and D; **P*<0.05, ***P*<0.01, ****P*<0.001, *****P* <0.0001; Quantitative analysis was calculated as the ratio of positive area /total area of tissue section; Quantitative data were shown as mean ± SEM; Statistical analysis in A and D was performed with Student’s t test.


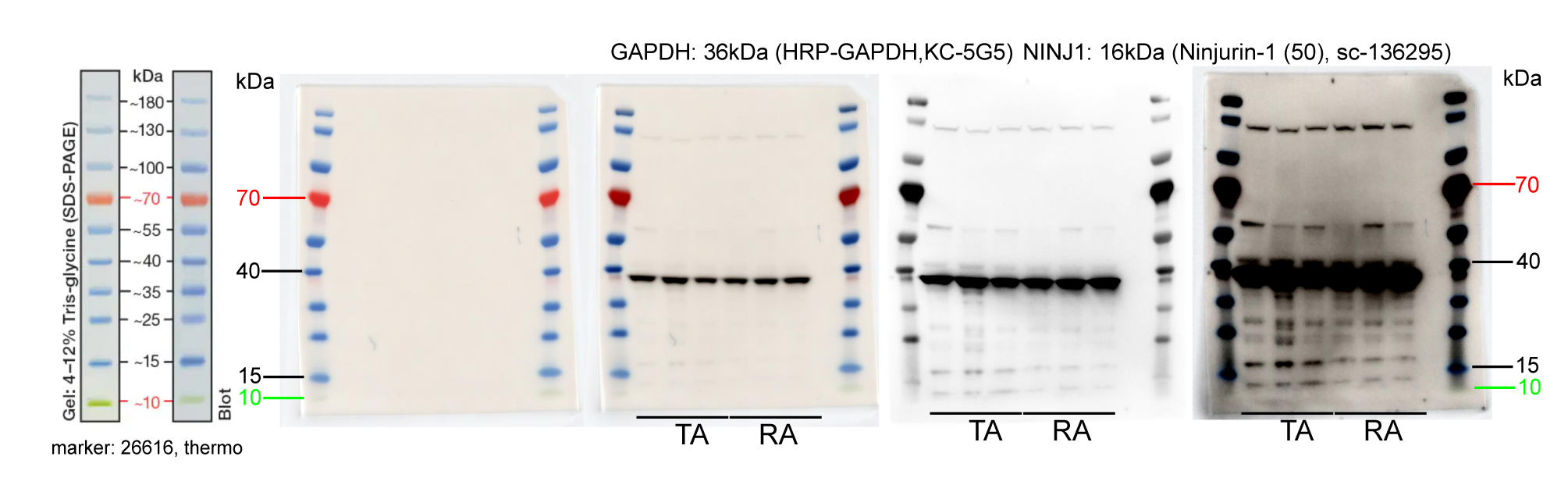


**Fig. S6.** **WB analysis for NINJ1 in TA and RA of human TAD tissue**

PageRuler Prestained Protein Ladder (#26616, Thermo Scientific) was used to indicate molecular weight of NINJ1(16 kDa, a cell surface protein) and GAPDH (36kDa, as loading control) in the same image. Ultrasensitive chemiluminescence imaging system (Amersham ImageQuant800) was used to detect the images of the original, uncropped blots. TA, tear area; RA, remote area.

##
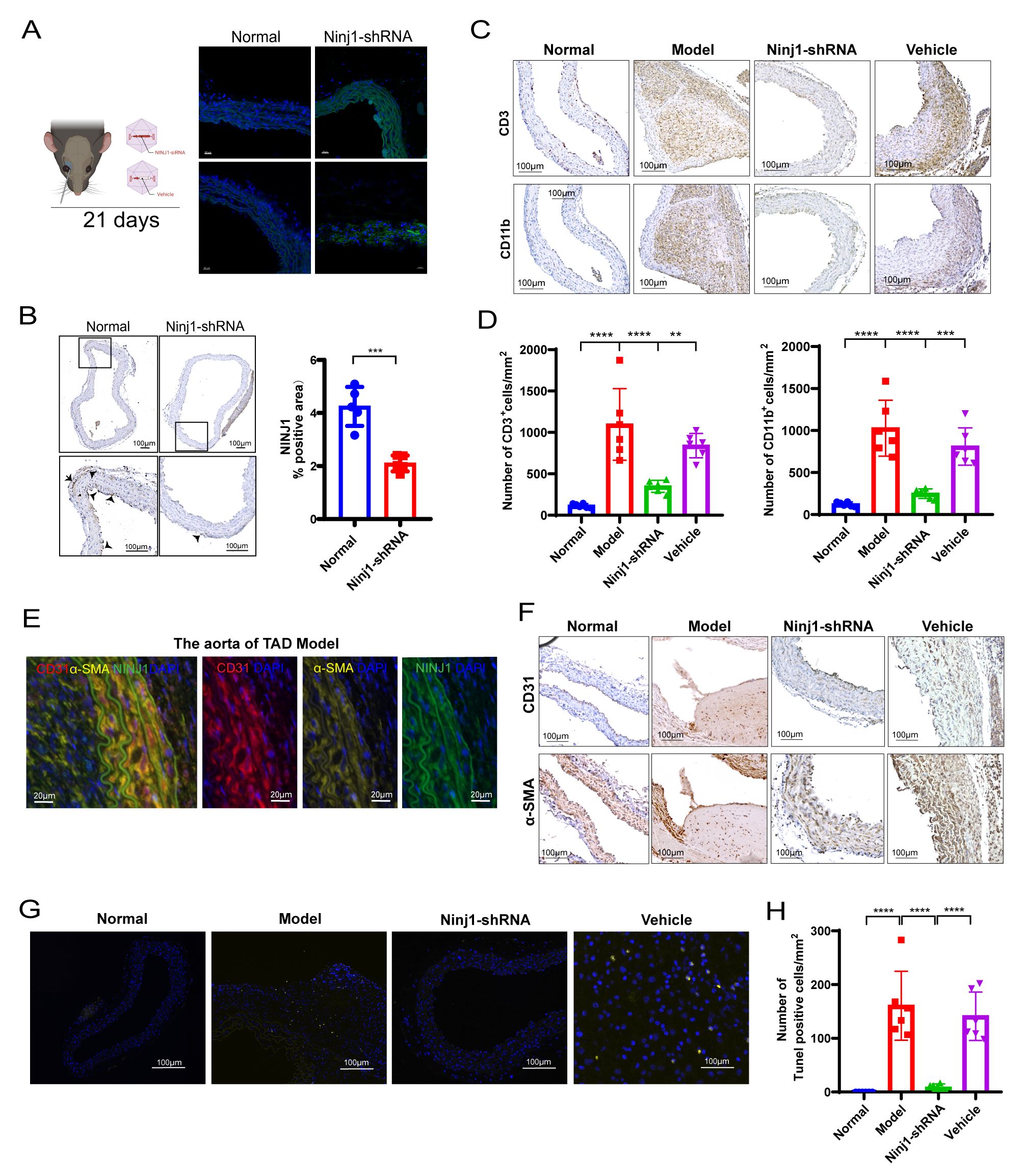
Fig. S7. AAV9-Ninj1-shRNA limited inflammation, tissue remodeling, and cell death

**A** GFP labeling confirmed successful AAV9 transduction**. B** Immunohistochemistry staining of NINJ1 between normal with or with Ninj1-shRNA treatment (n=6/group). Black arrows indicate NINJ1 positive points. **C** Immunohistochemistry staining of CD3 and CD11b in each group under different conditions. **D** Quantitative analysis of CD3 and CD11b (n=6, 5, 6 and 6, respectively). **E** Immunofluorescent staining of NINJ1, CD31, and α-SMA cells in aorta of TAD model**.** **F** Immunohistochemistry staining of CD31 and α-SMA in each group under different conditions. **G** Tunel staining in each group under different conditions. **H** Quantitative analysis of Tunel^+^ cells per mm^2^ (n=6, 5, 6 and 6, respectively) **P*<0.05, ***P*<0.01, ****P*<0.001, *****P* <0.0001; quantitative analysis of related positive area was calculated as the positive area /total area of tissue section, and analysis of number of cells in tissues was calculated as the ratio of a total number of positive points /the area of the entire section; quantitative data were shown as mean±SEM; Statistical analysis in B was performed with Student’s t test, and with One-way ANOVA with Tukey’s tests in D and H.


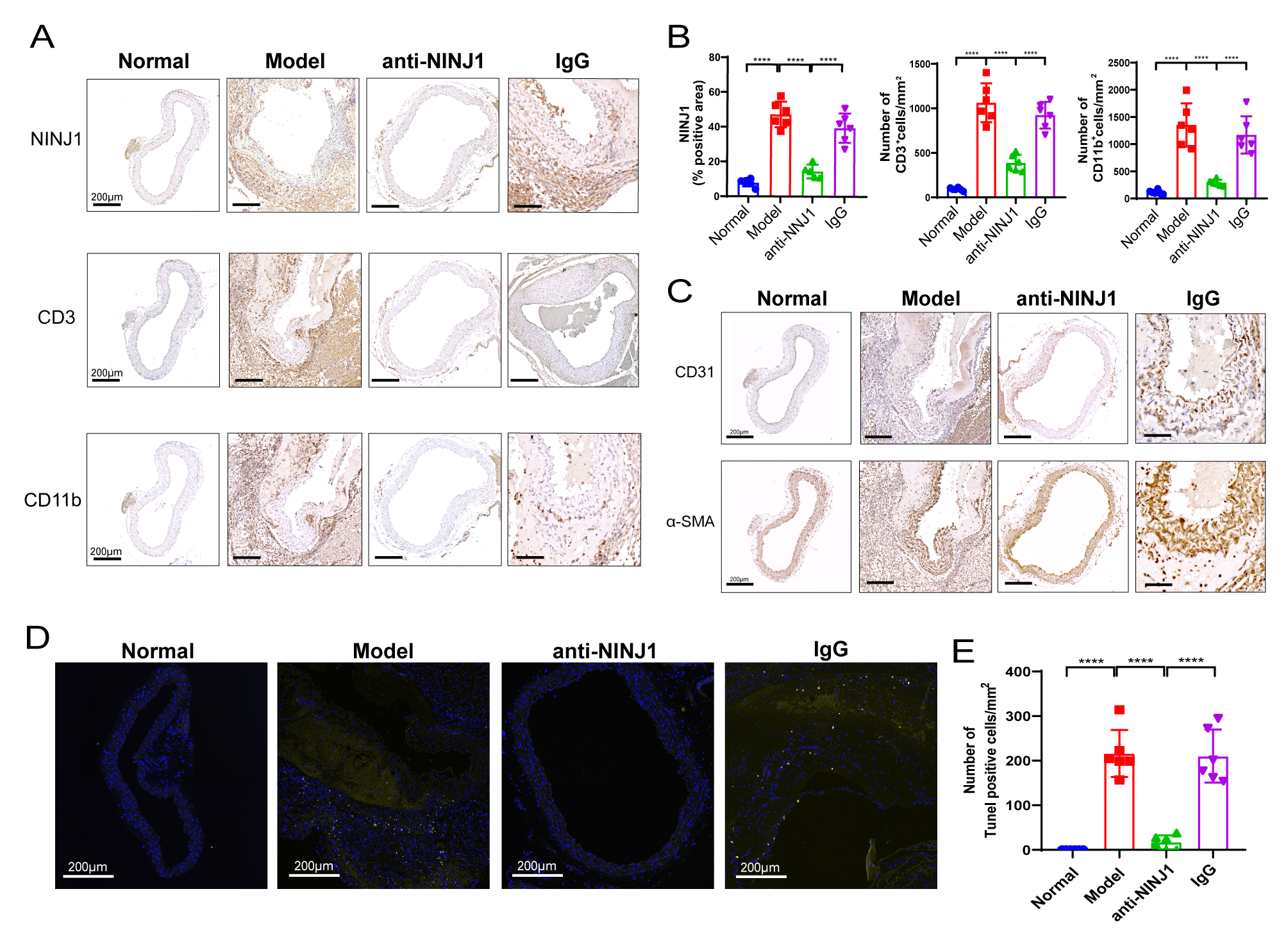


**Fig. S8.** **NINJ1-neutralization antibody limited inflammation, tissue remodeling, and cell death**

**A** Immunohistochemistry staining of NINJ1, CD3, and CD11b in each group under different conditions. **B** Quantitative analysis of CD3, CD11b, and NINJ1. **C** Immunohistochemistry staining of CD31 and α-SMA in each group under different conditions. **D** Tunel staining in each group under different conditions. **E** Quantitative analysis of Tunel^+^ cells per mm^2^. N=6 for each group in B and E; **P*<0.05, ***P*<0.01, ****P*<0.001, *****P* <0.0001; Quantitative analysis of related positive area was calculated as the positive area /total area of tissue section, and analysis of number of cells in tissues was calculated as the ratio of a total number of positive points /the area of the entire section; Quantitative data were shown as mean ± SEM; Statistical analysis in B and E was performed with One-way ANOVA with Tukey’s tests.


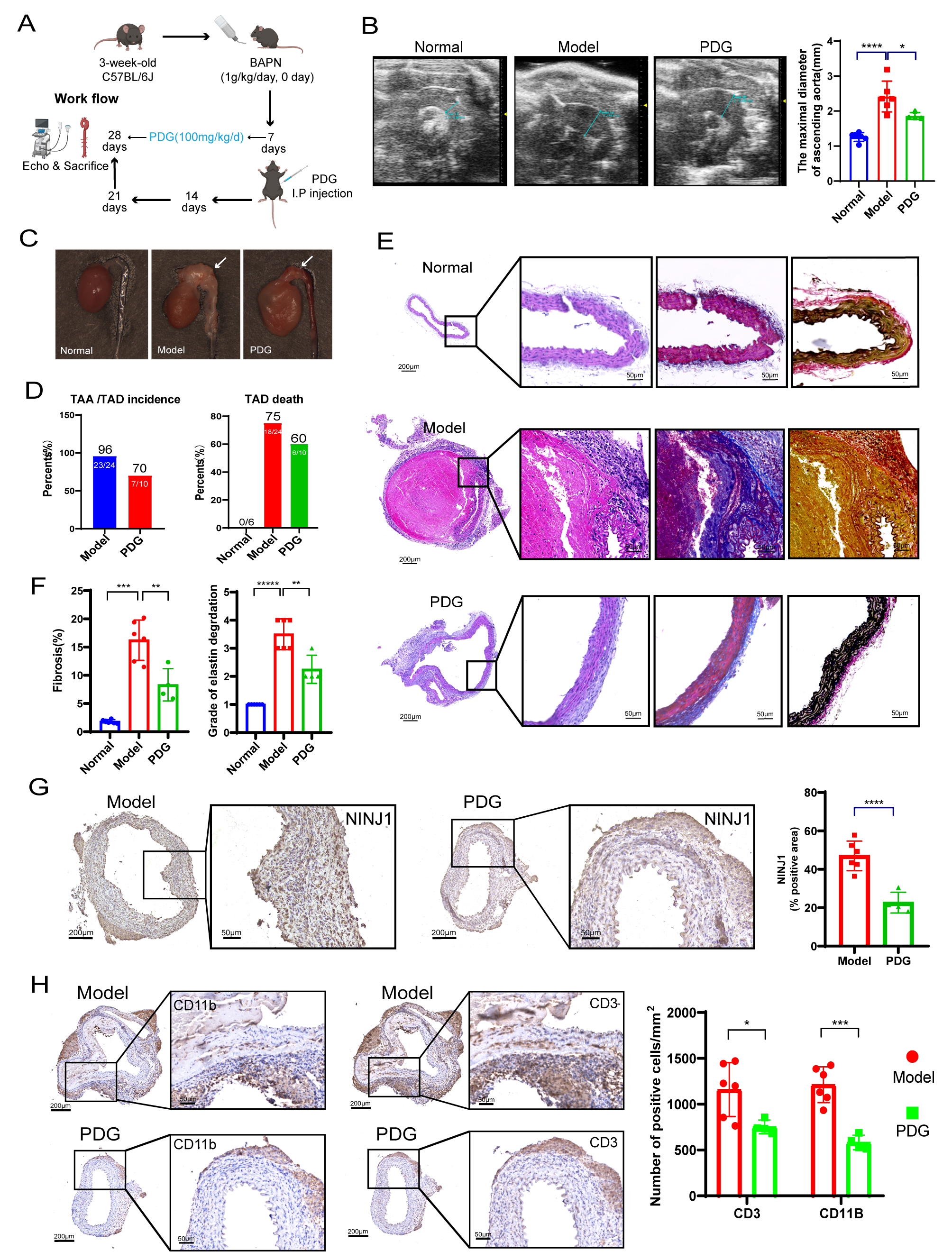


**Fig. S9. PDG inhibited BAPN-induced TAD formation**

**A** Workflow for PDG treatment experiments. **B** Ultrasonographic examination of the thoracic aorta maximal diameter in each group of surviving mice at the end of the experiment (n=6,6, and 4, respectively). **C** Representative photographs of aortas in each group. **D** The incidence and mortality of TAD in each group at the end time of the experiment. **E** Representative H&E, Masson, and EVG staining of the mouse thoracic aortas in each group. **F** Quantitative analysis of thoracic aortic fibrosis area, and grade of the elastin degradation in each group (n=6, 6 and 4, respectively). **G** Representative images and quantitative analysis of immunohistochemistry for NINJ1 in Model and PDG treatment mice. And the quantitative analysis of NINJ1((n=6 and 4, respectively). **H** Representative images and quantitative analysis of immunohistochemistry for CD3 and CD11b in Model and PDG treatment mice. ((n=6 and 4, respectively). **P*<0.05, ***P*<0.01, ****P*<0.001, *****P* <0.0001; Quantitative analysis of related positive area was calculated as the positive area /total area of tissue section, and analysis of number of cells in tissues was calculated as the ratio of a total number of positive points /the area of the entire section; Quantitative data were shown as mean ± SEM. Statistical analysis in B and F was performed with One-way ANOVA with Tukey’s tests, and Chi-sqare test in D, and Student’s t test in G, respectively.
