# Supplementary material for: Tomo-seq identifies NINJ1 as a potential target for anti-inflammatory strategy in thoracic aortic dissection
Source: BMC Med. 2023 Oct 20;21:396. doi: 10.1186/s12916-023-03077-1 (PMC10588060; doi:10.1186/s12916-023-03077-1)
Supplement: Supplementary file 3 — Additional file 3: Table S1. Main clinical characteristics of patients with TAD. Table S2. RNA concentration of different total slices thickness. Table S3. RNA concentration of ten consecutive sections with a thickness of 10µm. Table S4. RNA concentration of twenty consecutive sections with a thickness of 5µm. Table S5. Reads statistics results of TAD Cryosectioning. Table S6. Mapping of TAD Cryosectioning. [file 12916_2023_3077_MOESM3_ESM.docx]

Supplementary Tables

| **Characteristics** | **TAD(n=8)** |
| --- | --- |
| **Age (y)** | 61.75 ± 12.48 |
| **Men** | 6(60%) |
| **BMI** | 24.21± 2.99 |
| **Hypertension** | 10(100%) |
| **Diabetes mellitus** | 0 |
| **History of smoking** | 4(40%) |
| **Aortic diameter (mm)**  **Stanford classification** | 43.75 ± 6.09  Type A |

**Table S1.** Main clinical characteristics of patients with TAD.

Data are expressed as a number (percent) or as the mean ± standard error of the mean (SEM)

**Table S2. RNA concentration of different total slices thickness**

| **Patient** |  | **The total slices thickness (μm)** | **RNA concentration (ng/µl)** | **Total volume (μl)** |
| --- | --- | --- | --- | --- |
| Patient1 |  | 100 | 17.4 | 20 |
| Patient2 |  | 100 | 14.9 | 20 |
| Patient3 |  | 100 | 16.8 | 20 |
| Patient1 |  | 150 | 11.6 | 20 |
| Patient2 |  | 150 | 14.9 | 20 |
| Patient3 |  | 150 | 12.5 | 20 |
| Patient1 |  | 200 | 7 | 20 |
| Patient2 |  | 200 | 9.3 | 20 |
| Patient3 |  | 200 | 9.8 | 20 |
| Patient1 |  | 300 | 14.1 | 20 |
| Patient2 |  | 300 | 8 | 20 |
| Patient3 |  | 300 | 11.6 | 20 |

**Table S3. RNA concentration of ten consecutive sections with a thickness of 10µm**

| **Patient** |  | **The single-slice thickness**  **(μm)** | **Slices** | **RNA concentration (ng/µl)** | **260/280** | **260/230** | **Total volume**  **(μl)** |
| --- | --- | --- | --- | --- | --- | --- | --- |
| Patient1 |  | 10 | 10 | 4.6 | 2.00 | 0.51 | 20 |
| Patient1 |  | 10 | 10 | 6.5 | 1.98 | 0.33 | 20 |
| Patient2 |  | 10 | 10 | 8.9 | 1.80 | 0.71 | 20 |
| Patient2 |  | 10 | 10 | 5.8 | 2.03 | 0.31 | 20 |
| Patient3 |  | 10 | 10 | 4.4 | 1.86 | 0.37 | 20 |
| Patient3 |  | 10 | 10 | 6.9 | 1.81 | 0.71 | 20 |

**Table S4. RNA concentration of twenty consecutive sections with a thickness of 5µm**

| **Patient** | **The single-slice thickness**  **(μm)** | **Slices** | **RNA concentration (ng/µl)** | **260/280** | **260/230** | **Total volume （μl）** |
| --- | --- | --- | --- | --- | --- | --- |
| Patient1 | 5 | 20 | 13.8 | 1.80 | 0.34 | 20 |
| Patient1 | 5 | 20 | 12.2 | 1.96 | 0.63 | 20 |
| Patient2 | 5 | 20 | 16.4 | 1.92 | 0.86 | 20 |
| Patient2 | 5 | 20 | 14.7 | 2.10 | 0.93 | 20 |
| Patient3 | 5 | 20 | 15.1 | 1.89 | 0.92 | 20 |
| Patient3 | 5 | 20 | 11.5 | 1.89 | 0.93 | 20 |

**Table S5. Reads statistics results of TAD Cryosectioning**

| **Sample Name** | **Clean**  **Reads** | **Clean**  **bases** | **Read length (bp)** | **Q20(%)** | **GC(%)** |
| --- | --- | --- | --- | --- | --- |
| AD1 | 67,767,092 | 10,165,063,800 | 150 | 97.60% | 48.12% |
| AD2 | 68,195,782 | 10,229,367,300 | 150 | 97.62% | 48.65% |
| AD3 | 68,183,212 | 10,227,481,800 | 150 | 97.79% | 48.49% |
| AD4 | 67,892,720 | 10,183,908,000 | 150 | 97.50% | 47.69% |
| AD6 | 68,823,922 | 10,323,588,300 | 150 | 97.63% | 48.56% |
| AD7 | 67,843,832 | 10,176,574,800 | 150 | 97.64% | 49.36% |
| AD8 | 67,514,342 | 10,127,151,300 | 150 | 97.56% | 49.20% |
| AD9 | 67,744,692 | 10,161,703,800 | 150 | 97.60% | 48.88% |
| AD10 | 68,524,638 | 10,278,695,700 | 150 | 97.59% | 49.08% |
| AD11 | 67,961,468 | 10,194,220,200 | 150 | 97.62% | 49.48% |
| AD12 | 68,234,740 | 10,235,211,000 | 150 | 97.63% | 48.62% |
| AD13 | 67,565,820 | 10,134,873,000 | 150 | 97.74% | 49.11% |
| AD14 | 67,905,520 | 10,185,828,000 | 150 | 97.55% | 49.12% |
| AD15 | 68,116,236 | 10,217,435,400 | 150 | 97.56% | 49.05% |
| AD17 | 67,285,082 | 10,092,762,300 | 150 | 97.57% | 49.30% |
| AD18 | 69,079,310 | 10,361,896,500 | 150 | 97.42% | 48.98% |
| AD19 | 68,048,038 | 10,207,205,700 | 150 | 97.75% | 47.98% |
| AD20 | 68,040,780 | 10,206,117,000 | 150 | 97.68% | 49.00% |
| AD21 | 69,141,784 | 10,371,267,600 | 150 | 97.65% | 50.09% |
| AD22 | 68,778,908 | 10,316,836,200 | 150 | 97.78% | 48.94% |
| AD23 | 68,162,152 | 10,224,322,800 | 150 | 97.66% | 48.64% |
| AD24 | 67,905,244 | 10,185,786,600 | 150 | 97.54% | 48.83% |
| AD25 | 68,254,538 | 10,238,180,700 | 150 | 97.59% | 48.97% |
| AD26 | 67,609,184 | 10,141,377,600 | 150 | 97.79% | 48.78% |
| AD27 | 68,049,264 | 10,207,389,600 | 150 | 97.68% | 48.78% |
| AD29 | 68,927,964 | 10,207,389,600 | 150 | 97.64% | 48.81% |

**Table S6. Mapping of TAD Cryosectioning**

| **Sample name** | **Read number** | **Read uniquely mapping rate** |
| --- | --- | --- |
| AD1 | 33883546 | 92.15% |
| AD2 | 34097891 | 92.52% |
| AD3 | 34091606 | 93.40% |
| AD4 | 33946360 | 92.67% |
| AD6 | 34411961 | 94.59% |
| AD7 | 33921916 | 93.45% |
| AD8 | 33757171 | 93.19% |
| AD9 | 33872346 | 94.25% |
| AD10 | 34262319 | 94.22% |
| AD11 | 33980734 | 94.29% |
| AD12 | 34117370 | 93.86% |
| AD13 | 33782910 | 93.99% |
| AD14 | 33952760 | 94.62% |
| AD15 | 34058118 | 93.85% |
| AD17 | 33642541 | 94.53% |
| AD18 | 34539655 | 95.15% |
| AD19 | 34024019 | 93.70% |
| AD20 | 34020390 | 93.55% |
| AD21 | 34570892 | 94.48% |
| AD22 | 34389454 | 93.53% |
| AD23 | 34081076 | 93.81% |
| AD24 | 33952622 | 94.40% |
| AD25 | 34127269 | 95.10% |
| AD26 | 33804592 | 93.37% |
| AD27 | 34024632 | 94.76% |
| AD29 | 34463982 | 94.79% |

The Fastq data was compared to the human genome using STAR (v2.5.3a), and then the gene expression amount TPM (transcript per million) was calculated using RSEM (v1.2.31), using the gene annotation GENCODE v31.The sequencing data comparison results showed that the alignment rate of each sample (layer) was above 90%.
